# Contributions of transcription and mRNA decay to gene expression dynamics of fission yeast in response to oxidative stress

**DOI:** 10.4161/rna.29196

**Published:** 2014-07-09

**Authors:** Samuel Marguerat, Katherine Lawler, Alvis Brazma, Jürg Bähler

**Affiliations:** 1 Department of Genetics, Evolution & Environment and UCL Cancer Institute; University College London; London, UK; 2 European Molecular Biology Laboratory; EMBL-EBI; Wellcome Trust Genome Campus; Hinxton, UK

**Keywords:** fission yeast, mathematical modeling, mRNA turnover, oxidative stress, RNA Polymerase II, post-transcriptional control, gene regulation, Atf1, ribosome biogenesis

## Abstract

The cooperation of transcriptional and post-transcriptional levels of control to shape gene regulation is only partially understood. Here we show that a combination of two simple and non-invasive genomic techniques, coupled with kinetic mathematical modeling, affords insight into the intricate dynamics of RNA regulation in response to oxidative stress in the fission yeast *Schizosaccharomyces pombe*. This study reveals a dominant role of transcriptional regulation in response to stress, but also points to the first minutes after stress induction as a critical time when the coordinated control of mRNA turnover can support the control of transcription for rapid gene regulation. In addition, we uncover specialized gene expression strategies associated with distinct functional gene groups, such as simultaneous transcriptional repression and mRNA destabilization for genes encoding ribosomal proteins, delayed mRNA destabilization with varying contribution of transcription for ribosome biogenesis genes, dominant roles of mRNA stabilization for genes functioning in protein degradation, and adjustment of both transcription and mRNA turnover during the adaptation to stress. We also show that genes regulated independently of the bZIP transcription factor Atf1p are predominantly controlled by mRNA turnover, and identify putative *cis*-regulatory sequences that are associated with different gene expression strategies during the stress response. This study highlights the intricate and multi-faceted interplay between transcription and RNA turnover during the dynamic regulatory response to stress.

## Introduction

Gene expression is dynamically tuned to environmental factors, linking genotype with phenotype. Historically, regulation of transcription has attracted most attention and efforts. However, gene expression is also regulated at multiple post-transcriptional levels. Global studies examining correlations of features diagnostic of transcriptional or post-transcriptional regulation have afforded insight into transcriptome expression.^1^^-^^4^ Nevertheless, the cooperation between transcriptional and post-transcriptional regulation and their relative contributions to gene expression in response to internal or external factors are still not fully understood.

Among the post-transcriptional layers of regulation, mRNA turnover is known to contribute to the control of mRNA abundance. The regulation of mRNA decay has been examined at genome-wide levels.^5^^-^^7^ Transcript half-lives show a large dynamic range and can vary substantially between conditions and organisms. In addition, genes with related functions sometimes share similar decay rates and coordinate their turnover, leading to the concept of ‘decay regulons’.^8^^-^^10^ Besides mature protein coding transcripts, RNA degradation rates have also been shown to vary greatly among classes of long non-coding RNAs,^11^^,^^12^ and introns.^13^ Moreover, regulation of mRNA turnover in response to environmental cues can strongly affect the particular gene expression kinetics. For instance, budding yeast uses rapid mRNA de-stabilization to achieve a strong, transient upregulation of stress genes for a rapid response to oxidative stress.^14^ For long-term adaptation to osmotic stress, however, both budding yeast and fission yeast tend to use transcript stabilization for sustained transcript upregulation.^14^^-^^16^ These data demonstrate that regulation of mRNA decay is instrumental in shaping gene expression profiles, resulting in distinct kinetics of gene expression.

These studies did not directly address, however, how regulation at the levels of transcription and transcript turnover is integrated. This question requires approaches to separate the respective contributions of transcription and mRNA decay to gene expression profiles. The contribution of transcription to the DNA damage response in human T-cells has been modeled based on mRNA expression and decay data.^17^ Alternatively, transcription rates and mRNA expression levels can be measured and used to deduce the contribution of transcript decay to gene expression. Global transcription rates can be estimated by determining the activity of transcriptionally engaged RNA polymerases using genomic run-on analysis, by measuring RNA polymerase II (Pol II) occupancy, by detecting newly transcribed RNA after metabolic labeling,^6^^,^^18^ or by comparing pre-mRNA to mature mRNA levels.^19^ Such studies have revealed gene expression strategies characterized by the coordinated regulation of transcription and mRNA turnover. This regulation can be global, as in the case of large metabolic reprogramming, or it can be more specific and control only genes belonging to defined functional categories.^13^^,^^18^^,^^20^^-^^22^ Moreover, the strength of the perturbation can affect the regulatory strategy applied by the cell.^23^

The mathematical approaches used for these studies to disentangle the respective contributions of transcription and mRNA decay have assumed that the transcriptome is shifting between a series of steady-states, even during dynamic responses. Comparisons between transcription rate and mRNA abundance at steady-state have been used to identify stress response-modulated mRNA decay rates in budding yeast,^20^ in human lung carcinoma cells,^24^ and in tobacco.^25^ A step-wise linear approach to modeling temporal profiles in response to stress has been applied to nuclear run-on time courses in budding yeast, avoiding steady-state assumptions but giving a series of estimates for mRNA decay rates between two adjacent time points.^26^^,^^27^ Transient up- and downregulation of mRNA abundance and subsequent recovery has been modeled as rapid transitions between steady-states,^14^^,^^28^ or using a two-sigmoid ‘impulse’ and recovery model to describe the shape and timing of a transient gene expression response.^29^^,^^30^ A dynamic model of transcript production and degradation has been used to further explain global differences in peak times and peak abundances between pre-RNA and mRNA, and to infer time-dependent transcript production and decay rates for 12 individual genes from mRNA and pre-RNA qPCR profiles in human epithelial cells.^19^

Here, we apply non-invasive experimental and computational approaches to identify gene expression strategies during a dynamic response to oxidative stress, using the fission yeast *Schizosaccharomyces pombe* as a simple model system. We acquire genome-wide estimates of transcription rates and transcript levels by measuring Pol II occupancy and mRNA expression signals, respectively, on the same microarray platform. We then extract the contribution of mRNA decay from these data sets using a model selection approach to compare simple kinetic models that do not require steady-state transcript levels. Our study reveals that on top of the dominant transcriptional control, post-transcriptional regulation participates in global regulation of gene expression and helps to shape the rapid and dynamic response. In addition, specific gene classes make selective use of controlled mRNA turnover to achieve specialized gene expression patterns.

## Results and Discussion

### Global measurements of mRNA levels and transcription rates

To understand how transcription and mRNA turnover contribute to dynamic gene expression profiles during adaptation to oxidative stress, we globally measured mRNA levels and transcription rates from a high-resolution time course, encompassing 12 time points over 2 h, after addition of hydrogen peroxide (H_2_O_2_) to proliferating fission yeast cells (Fig. S1A; Table S1). Global transcript levels were measured for each time point by hybridizing cDNA to DNA microarrays.^31^ In parallel, genome-wide data of Pol II occupancy were obtained for the same samples by Pol II chromatin immunoprecipitation followed by hybridization onto the same microarray platform (Pol II ChIP-chip; Fig. S1B). These two data sets were then used as inputs for a mathematical model to extract the contribution of mRNA decay to gene expression kinetics. This combined approach has the advantage of being non-invasive, by omitting the need for chemicals, mutants or extensive treatments that are typically required for global measurements of mRNA decay or transcription rates.

We used the Pol II occupancy data as estimates for transcription rates. This approach is equivalent at the single-gene level to a nuclear run-on.^32^ Globally, Pol II occupancy generally correlates with transcript levels,^2^^,^^33^ and with transcription rates as measured by run-on^34^ or metabolic labeling.^18^ In order to validate this approach further, we confirmed that the dynamic range of Pol II ChIP-chip could detect variations across the entire range of transcription rates, by examining the amplitude of variation in Pol II occupancy. We used a value defined for each gene as the ratio between any time points with the highest and lowest Pol II occupancy values (Maximum Fold Changes in Pol II occupancy: MFC_POL_). Fig. S2 shows MFC_POL_ as a function of Pol II occupancy values from cells immediately before stress induction. Maximum changes above 2-fold (log2 MFC_POL_ = 1) could be detected across the entire distribution of initial values for Pol II occupancy, indicating that the method has a large dynamic range and is not affected by saturation effects. In addition, genes with robustly induced or repressed transcription were both above the 2-fold threshold, showing that the assay has no directional bias (Fig. S2, orange and green dots, respectively). Taken together, these data indicate that measurement of Pol II occupancy is a robust approach to estimate dynamic changes in transcription rates.

The estimation of transcription rates could be confounded in certain cases, because Pol II transcription can be regulated at the level of transcription elongation, leading to Pol II stalling at, or downstream of, promoters^35^ However, our array design uses probes in exonic regions located near the 3′ ends of genes,^31^ which minimizes the probability that our measurements are affected by stalled Pol II complexes on promoters or intronic regions. Accordingly, the distance between each gene microarray probe and the nearest transcription start site (TSS) was not correlated with their MFC_POL_ (R_Pearson_ = -0.007). Moreover, ordering genes according to their probe distance from the nearest TSS and sorting them into bins, did not detect lower MFC_POL_ in genes with probes close to TSS as expected in the case of translational regulation or stalled Pol II at or near promoters (Fig. S3). On the contrary, genes with probes close to TSS showed significantly higher MFC_POL_, probably due to their enrichment for ribosomal proteins, a functional class strongly regulated during stress response (see below). Any bias due to Pol II stalled inside coding regions is more difficult to control for; in order to minimize the possible impact of such a bias on our conclusions, the mathematical modeling approach to determine the contribution of mRNA decay also included parameters that take into account possible variations in effective baseline Pol II occupancy for each gene which could result from inactive Pol II stalled inside coding regions^34^ or from antisense transcription.^33^ Finally, our analytical approach did not use Pol II occupancy as an absolute measure of transcription rates but as an estimate of changes relative to pre-stress transcription rates for each gene. Taken together, these results indicate that the experimental and computational design applied in this study minimizes potential confounding signals due to Pol II molecules stalled at the 5′ end of genes.

### Early transcriptional response controls mRNA levels during oxidative stress

Regulation of transcription is expected to play an important role for rapid adaptation of gene expression to changing conditions. In order to test this assumption, we first compared the relationship between changes in transcription rates and changes in transcript levels in our stress-response data sets. To this end, we calculated maximum fold changes for the transcript expression data (MFC_EXP_) and Pol II occupancy data (MFC_POL_). These values are measures of the amplitude of changes in mRNA levels and in transcription during the stress response.

Notably, MFC_EXP_ and MFC_POL_ were positively correlated with each other (r_Pearson_ = 0.65, P_Pearson_ < 2.2 10^−16^, Fig. S4A), indicating that the overall variation in transcription rates is an important factor determining changes in transcript levels during stress adaptation. To globally visualize this result, genes with an MFC_EXP_ above 2-fold were clustered by their mRNA expression and Pol II occupancy profiles (Fig. 1). This analysis revealed that transcript expression and Pol II occupancy followed similar kinetics for most genes, indicating that transcription is vital for modulating the timing of gene regulation during rapid adaptation to stress. In order to substantiate this observation, we calculated the correlation between Pol II occupancy and transcript expression for each gene with an MFC_EXP_ > 2-fold. This analysis revealed a median coefficient of correlation of 0.7 (Fig. S4B), confirming that regulation of transcription is a dominant determinant of the transcript expression kinetics during the stress response. In addition, adaptation to environmental stress was also dominated by a transcriptional response in budding yeast, suggesting an evolutionary conserved gene expression strategy.^6^^,^^26^^,^^36^^,^^37^

**Figure:**
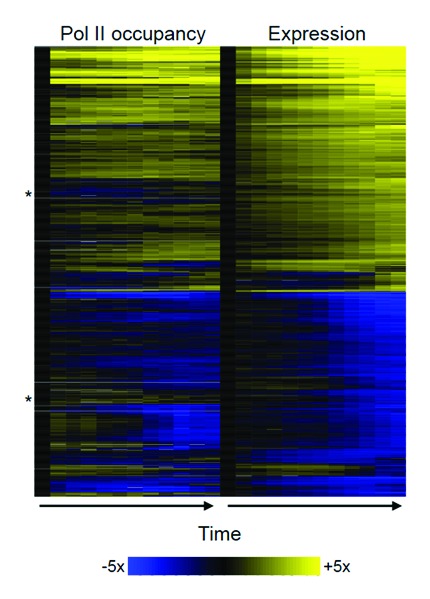
**Figure 1.** Dynamic changes in gene expression largely reflect changes in Pol II occupancy during oxidative stress. Genes with large regulation in Pol II occupancy and transcript expression data (MFC_EXP_ and MFC_POL_ > 2-fold, see main text) were hierarchically clustered (Pearson correlation) using GeneSpring GX7 software (Agilent). Clustering results were plotted as a heat-map, where upregulated genes are shown in yellow and downregulated genes in blue as indicated at the bottom (gray: no data). Example sub-clusters showing signs of dominant post-transcriptional regulation are marked by asterisks.

We then assessed the timing of transcriptional induction. To this end, we compared the variance of the fold changes in transcription between time points (Fig. S5). This analysis revealed that the biggest amount of variation occurred within the first 5 min after stress induction, indicating that a short time window immediately after stress exposure is key to launch the transcriptional response. Taken together, these data demonstrate that global and rapid transcriptional regulation can lead to swift adaptation of gene expression in response to changing conditions. However, these data also show that transcriptional changes do not fully explain the expression profiles, leaving scope for substantial post-transcriptional regulation.

### Modeling the changes in mRNA turnover during oxidative stress

We next evaluated the contribution of post-transcriptional regulation, and in particular control of mRNA decay. We first examined whether adjustment of mRNA turnover is required at all to explain the rapid changes in gene expression during stress. To this end, we applied mathematical modeling of the relative changes in transcript levels and transcription rates determined from the microarray data.

We applied two kinetic models for alternative scenarios. First, we assumed that transcript levels can be explained by a model in which transcription rates vary during the stress response while degradation rates are kept constant after stress. This ‘constant’ model assumed that the measured change in transcript abundance y(t) resulted from a time-varying transcription rate [measured as relative values f(t)] and a degradation rate which is proportional to concentration with decay rate constant k. The solution, given absolute measurements of mRNA abundance with production rate P(t), was previously described:^9^^,^^26^^,^^37^

$$[RNA]=\int_{0}^{t}P(t')e^{k(t'-t)}dt'+\text{​}{\left[RNA\right]}_{0}e^{-kt}$$

Given relative measurements of transcription rate [f(t)] and mRNA abundance [y(t)]:

$$y(t)=A\int_{}^{t}f(t')e^{k(t'-t)}dt'+\text{​}B+\text{​}Ce^{-kt}$$

where A, B, C are gene-specific constants (Experimental procedures). A special case (A = 0) describes an exponential approach of mRNA abundance due to an instantaneous change in decay rate at the start of the time course (t = 0).

The second model describes an alternative scenario in which an additional instantaneous mRNA stabilization or destabilization event happens at some time during the stress response (Experimental procedures, Equation 3). This ‘switch’ model extends the ‘constant’ model by allowing piece-wise first-order decay, and was designed to capture changes in transcript decay rates while restricting the number of additional parameters to be fitted in the model:

$$y(t)=A\int_{}^{t}f(t')e^{k_{1}(t'-t)}dt'+\text{​}B+\text{​}Ce^{-k_{1}t};\;t\leq t_{switch}$$

$$y(t)=A\int_{}^{t}f(t')e^{k_{2}(t'-t)}dt'+\text{​}B\frac{k_{1}}{k_{2}}+\text{​}Ge^{-k_{2}t};\;t>t_{switch}$$

where constant G was chosen to satisfy continuity at t_switch_. Both models were fitted to the 3769 fission yeast genes with complete data for all time points. The best-fit model was selected for each gene using a goodness-of-fit criteria adjusted for number of parameters and a threshold for the minimum detected change in decay rate during the stress response (Experimental procedures). Using this approach, 3257 genes were assigned to either model (Table S2). To verify that the ‘constant’ model is an informative initial model for this time course, the goodness-of-fit distribution was compared before and after randomizing the association between transcription and expression profiles (Fig. S6A-D). The robustness of ‘switch’ model assignment was investigated by simulation of additional noise in the measured variables (Fig. S6E,F).

### Regulation of mRNA turnover during oxidative stress

In order to investigate the respective contribution of transcription and mRNA decay on individual mRNA levels, each gene was first assigned to one of the two mRNA decay models (‘constant’ or ‘switch’), and then grouped according to expression (both models) and transcription kinetics (‘constant’ model only) using the time series clustering software SplineCluster.^38^

The expression profiles of 2384 genes (73.2%) were best explained by the ‘constant’ model, and these were further split into 42 clusters (Clusters 1c-42c; Figure 2A; Fig. S7A). This finding suggests that a majority of transcripts show no, or little, changes in their stability after stress induction. Importantly, while these genes do not require any regulation in mRNA turnover during the time course to explain their expression profiles, it is still possible that mRNA turnover is modulated coherently with, and on top of, transcriptional control at the start of the time course (t = 0; initial steady-state). Our modeling approach permits further investigation of this possibility, because it predicts that an instantaneous change in transcription and/or turnover rates at the very beginning of the time course would lead to transcripts reaching a new steady-state with exponential kinetics (Experimental procedures). We exploited this characteristic as a complementary approach to test whether some transcripts undergo early stabilization or destabilization events. We could identify 910 transcripts showing exponential approaches to new steady-states, indicating that early and rapid changes in either transcription and/or turnover rates are important to shape gene expression during stress. These genes were grouped into 10 clusters (Clusters 1e-10e; Figure 2B; Fig. S7D). Interestingly, clusters 1e to 4e showed increasing transcript levels with exponential approach behavior. Comparison of transcription rates and transcript levels between the initial and final steady-state (after the exponential transition) showed the signature of an early stabilization of these transcripts (Experimental procedures). These data indicate that, on top of transcriptional control, the first minutes of the stress response can see concordant regulation of mRNA turnover.

**Figure:**
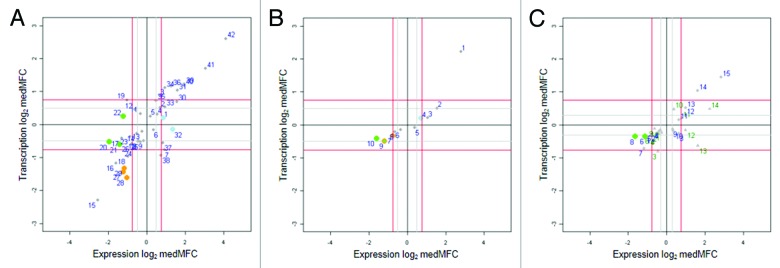
**Figure 2.** Multiple regulatory patterns involve modulation of transcription and/or mRNA turnover. Genes with good fits to one of three mathematical models are classified by Bayesian hierarchical clustering. The resulting clusters are plotted as a function of their median maximum-fold change values for expression (medMFC_EXP_) and for Pol II occupancy (medMFC_POL_). Orange dots represent clusters enriched for ribosomal proteins. Blue dots represent clusters regulated mainly at the mRNA stability level. Green dots are clusters enriched for genes of the Ribi regulon. (**A**) Clusters derived from genes assigned to the ‘constant’ model (1c-42c). (**B**) Clusters derived from genes assigned to the ‘constant’ model with exponential approach to a new steady-state (1e-10e). (**C**) Clusters derived from genes assigned to the ‘switch’ model (stabilized, 1ss-14ss, triangles; destabilized, 1ds-15ds, circles).

The expression profiles of another 873 genes (26.8%) were best explained by the ‘switch’ model, with mRNA turnover changing later during the time course in addition to transcriptional control. These genes were split into 25 clusters based on their expression profiles and whether the switch in transcript turnover led to mRNA stabilization (Clusters 1ss-14ss; triangles in Figure 2C; Fig. S7B) or mRNA destabilization (Clusters 1sd-15sd; circles in Figure 2C; Fig. S7C). However, when only considering genes with strong changes in mRNA levels (MFC_EXP_ > 2-fold), the proportion of genes explained by the ‘switch’ model dropped to 12.8%. We conclude that a majority of genes regulated during the stress response undergo either no or an early adjustment of decay rates, and that a minority of genes undergo delayed adjustment of mRNA turnover to regulate expression kinetics. Interestingly, similar early and late adaptation phases where transcription and degradation rates can be concomitantly regulated during stress response have also been described in the evolutionary distant budding yeast, hinting at the conservation of such regulatory strategies.^18^

### Early changes in transcription and mRNA turnover control gene expression for ribosomal proteins during oxidative stress

We further examined the genes from the ‘constant’ set for which our model predicted early or no regulation of mRNA turnover. Fourteen clusters, consisting of 605 genes, showed strong regulation in both transcript levels and transcription rates (Experimental procedures). Expression levels from six of these clusters were first downregulated relative to time point zero, while genes in seven clusters were induced (Fig. 2A, Table S3). These clusters consisted mostly of diverse stress response genes. Notably, three of the strongly repressed Clusters 27c, 28c, and 29c were highly enriched for transcripts encoding ribosomal proteins (Fig. 3A; Table S1). These transcripts are among the most stable in unstressed cells,^39^ which seems incompatible with rapid downregulation of their transcript levels during stress. We therefore hypothesized that ribosomal proteins are coordinately destabilized immediately after stress induction. To test this hypothesis, we first checked whether the ‘constant’ model predicts early mRNA destabilization for ribosomal protein genes (Fig. 2B). Indeed, Clusters 7e and 9e were significantly enriched for ribosomal proteins (P_Fisher_ < 10^−5^ and 10^−17^, respectively), providing evidence that these genes are regulated early by increased transcript turnover. To confirm this finding, we compared the half-lives of the mRNAs encoding ribosomal proteins calculated by our model to the half-live estimates measured in unstressed cells.^39^ Consistent with their rapid downregulation, transcripts in Clusters 27c-29c were significantly less stable after stress than before stress (P_Wilcox_ < 0.001, Fig. S8 and Table S4). In summary, this analysis strongly supports the idea that an immediate adjustment of transcription and transcript turnover rates at the time of stress induction is used to coordinately and rapidly downregulate the highly abundant transcripts encoding ribosomal proteins.

**Figure:**
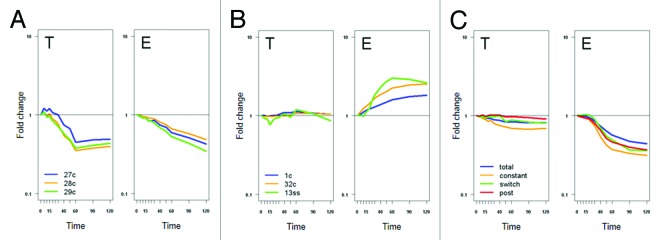
**Figure 3.** Specific gene categories use distinct expression strategies. Median transcription (T) and expression (E) profiles of selected gene groups with common expression strategies are plotted. Names of gene clusters are indicated in legend at lower left. These findings were validated by independent low-resolution time course experiments (Fig. S10). (**A**) Median expression profiles of genes encoding ribosomal proteins distributed in three clusters from the ‘constant’ model. (**B)** Median expression profiles of three clusters showing regulation mostly at the mRNA degradation level. Clusters 1c and 32c contain genes related to protein degradation, while cluster 13ss in enriched for *tf2* elements. (**C**) Median expression profiles of four subcategories of genes from the Ribi regulon: ‘total’, all genes in list; ‘constant’, genes assigned to ‘constant’ model showing transcriptional regulation; ‘switch’, genes assigned to ‘switch’ model; ‘post’, genes from ‘constant’ model showing little or no regulation at transcriptional level.

### mRNA stabilization dominates the stress regulation of specific functional gene categories

Even though transcriptional regulation was prevalent in our data, these results also suggested that the expression of a number of genes is dominated by post-transcriptional regulation (Fig. 1, asterisks). In order to search for gene expression profiles regulated mainly by mRNA turnover, we examined clusters characterized by high median MFC_EXP_ but small median MFC_POL_ (i.e., clusters where large changes in mRNA levels are associated with only marginal changes in transcription levels). In Figure 2A, these clusters are located away from the vertical zero axis (substantial change in expression), but close to the horizontal zero axis (marginal change in transcription). We identified the two Clusters 1c and 32c as being dominated by mRNA stabilization for their gene expression kinetics (Fig. 3B). As the modeled mRNA stability remained constant during the time course, the observed changes in transcript levels suggested stabilization of transcripts at the time of stress induction. The two clusters are associated with the GO term ‘Protein catabolic process’ (corrected P_Fischer_ = 5.1 x 10^−6^ and 6.4 x 10^−3^, respectively) and, for Cluster 1c only, with the GO term ‘Peptidase activity’ (corrected P_Fischer_ = 2.8 x 10^−4^). In order to further validate the dominant effect of regulated turnover of those transcripts, we asked whether the ‘constant’ model was detecting signs of early transcript stabilization (Fig. 2B). Indeed, Cluster 4e was enriched for genes associated with the GO term ‘Protein catabolic process’ and ‘Peptidase activity’ (corrected P_Fischer_ = 1 x 10^−4^ and 4 x 10^−4^, respectively) and showed a strong overlap with Cluster 1c (P_Fischer_ < 2.2 x 10^−16^). These findings thus confirmed our hypothesis that these genes are induced mostly by early transcript stabilization. Interestingly, stabilization of the proteasome during oxidative stress has been observed in budding yeast using different experimental approaches, confirming that regulatory strategies of genes with similar functions can be conserved during evolution.^14^^,^^27^ The *tf2* transposons also showed stabilization during stress but only later during the response (Cluster 13ss; Fig. S7B). Given the high sequence similarity among *tf2* transcripts, cross-hybridization may have occurred on the microarray. Nevertheless, these data indicate that at least one *tf2* transposon in the fission yeast genome is significantly regulated at the post-transcriptional level during oxidative stress. These observations are compatible with a model where the RNAi machinery controls a population of stress response genes at the nuclear pore by degrading newly generated transcripts in unstressed conditions but not during stress.^40^

### Ribosome biogenesis genes are regulated by complex program of transcription and mRNA turnover

Genes encoding ribosomal proteins and ribosome biogenesis factors belong to two tightly controlled regulons which underlie cell growth and stress protection.^41^^,^^42^ We showed that transcripts encoding ribosomal proteins are rapidly switched off after stress induction in a process regulated at both transcriptional and post-transcriptional levels. The ribosome biogenesis genes, also called Ribi regulon, are extensively regulated upon stress and growth arrest.^41^^-^^43^

To examine the regulatory strategies adopted by fission yeast to control expression of the Ribi regulon, we examined the profiles of 204 genes associated with ribosome biogenesis. As expected, the Ribi regulon was globally repressed during adaptation to stress, accompanied by a global repression of transcription (Fig. 3C, blue line). A majority of 133 genes was assigned to the ‘constant’ model (Clusters 20c to 22c), while 45 genes were characterized with delayed adjustment of mRNA turnover and were therefore assigned to the ‘switch’ model (Clusters 6sd and 8sd). We observed three groups of Ribi genes showing well defined expression strategies. First, expression of genes from Clusters 20c and 21c from the ‘constant’ set showed decreased expression accompanied by transcriptional repression (Fig. 3C, orange line). Interestingly, genes in Cluster 20c were significantly enriched for genes containing the RRPE regulatory sequence in their promoters. This sequence is important for transcriptional regulation of the Ribi regulon,^44^ indicating that genes under the control of the same regulatory sequence undergo coherent changes in transcription and gene expression levels. Second, genes from Clusters 6sd and 8sd showed decreased expression mediated by transcriptional repression and delayed adjustment of mRNA turnover occurring between 16 and 33 min after stress induction (Fig. 3C, green line). The first two classes represent 34.3% of the Ribi genes, revealing that mRNA decay plays a role in regulation of the Ribi regulon in fission yeast, besides the prevalent transcriptional regulation. Interestingly, a group of genes from the ‘constant’ set, representing ~10% of the Ribi genes, showed a clear decrease in expression levels but only marginal changes in transcription (Cluster 22c; Figure 3C, red line), indicating that their regulation is likely dominated by early changes in mRNA turnover. This hypothesis is consistent with the ‘constant’ model predicting early destabilization for these genes (Cluster 10e, Figure 2B). Taken together, these results indicate that the Ribi regulon makes use of multiple modes of regulation to tune the gene expression response in fission yeast.

### Regulation of Atf1p-independent genes during oxidative stress

Transcripts regulated during adaptation to different environmental stresses in fission yeast include a large group of Core Environmental Stress Response genes (CESR).^45^ The mitogen-activated protein kinase Sty1p and the bZIP transcription factor Atf1p control the expression of a group of CESR genes in 0.5 mM H_2_O_2_.^46^ However, this pathway, and especially Atf1p, only accounts for regulation of a fraction of CESR genes, suggesting the existence of additional regulators. These unknown regulators could involve either additional transcription factors and/or post-transcriptional control. To gain insight into this issue, we analyzed the contributions of transcription and mRNA stability to the regulation of genes whose expression is either dependent or independent of Atf1p. We first assigned the gene clusters defined in this study to three classes. The first class contains clusters of genes mainly dependent on Atf1p for stress regulation. These clusters were significantly enriched for genes loosing regulation in an *atf1* deletion strain.^45^ To avoid mis-categorization of clusters due to marginal regulation not detected by our cut-offs, we also required that the clusters show reduced amplitude in regulation in *atf1* mutant cells compared with wild-type controls (Fig. S9; Experimental Procedures). We named those clusters ‘Atf1p-dependent’. A second class, named ‘Atf1p-independent’, included genes whose regulation stayed unaffected in *atf1* deletion cells according to the criteria described above (Fig. S9). A third class contained genes which could not be assigned to any of the two categories according to these criteria.

Figure 4 shows the classification into ‘Atf1p-dependent’ or ‘Atf1p-independent’ clusters assigned to the ‘constant’ model; no clusters from the ‘switch’ model could be assigned to ‘Atf1p-dependent’ or ‘Atf1p-independent’ genes. Three ‘Atf1p-dependent’ clusters contained genes upregulated during stress (Clusters 31c, 41c, and 42c). These genes were characterized by substantial variations in expression and in transcription, consistent with Atf1p mediating regulation of these genes at the transcriptional level. In addition, Clusters 41c and 42c were significantly enriched for genes bound by Atf1p based on ChIP-chip experiments (corrected P_Fischer_ < 10^−8^),^47^ indicating that these genes are regulated by direct binding of Atf1p to their promoters. Three Atf1p-dependent clusters showed decreased expression during stress (Clusters 27c, 28c, and 29c). These clusters contain mainly ribosomal protein genes. Interestingly, no significant overlap was found with the Atf1p ChIP-chip data,^47^ raising the possibility that these genes are only indirectly repressed by Atf1p. In conclusion, our data show that Atf1p target genes can be split into distinct groups with coherent transcriptional and expression kinetics.

**Figure:**
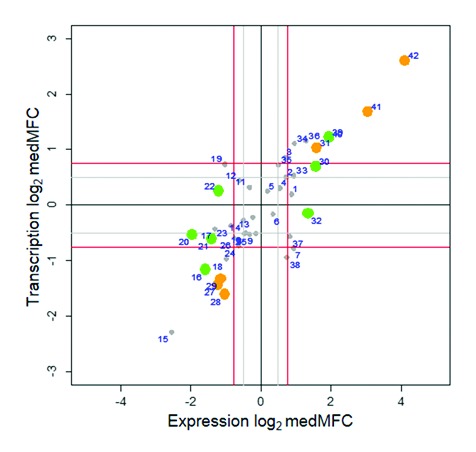
**Figure 4.** Atf1p-dependent and -independent genes show distinct expression strategies. Clusters from ‘constant’ model were plotted as a function of their medMFC_EXP_ and medMFC_POL_ (see legend Figure 2). Clusters were classified as ‘Atf1p-dependent’ (orange dots), ‘Atf1p-independent’ (green dots), or left unassigned (gray dots).

The ‘Atf1p-independent’ genes could be separated into the seven Clusters 16c, 20c, 21c, 22c, 30c, 32c, and 39c. Notably, most of these clusters were characterized by marginal changes in transcription, indicating that regulation of mRNA turnover is key in regulating the Atf1p-independent genes. This result explains why Atf1p has not been found to regulate these genes.^45^ However, two clusters showed substantial changes in transcription (Clusters 16c and 39c). Cluster 39c was significantly enriched in genes associated with amino acid metabolism (corrected P_Fischer_ < 10^−3^),^44^ and cell-cycle regulated genes (corrected P_Fischer_ < 10^−3^).^48^ Cluster 16c was also enriched for genes regulated during the cell cycle (corrected P_Fischer_ < 10^−4^). We conclude that the latter genes are regulated by an unknown transcription factor other than Atf1p, although the presence of cell cycle-regulated genes probably reflects cell-cycle arrest triggered during the stress response.^46^ In summary, our analysis demonstrates that genes regulated by Atf1p show a clear transcriptional response, while genes regulated independently of Atf1p are predominantly controlled by mRNA turnover.

### Sequence motifs associated with distinct regulatory strategies

RNA turnover can be regulated by RNA-binding proteins that recognize specific *cis*-regulatory sequence motifs. We therefore looked for enrichment in short nucleotide sequences upstream (5′UTR/promoter) and downstream (3′UTR/terminator) of open reading frames from genes regulated during oxidative stress. We first screened each cluster using the FIRE algorithm.^49^ We identified two conserved motifs in the 5′UTR/promoter regions of two distinct groups of clusters. The first motif is the transcription factor binding site called CRE which was enriched in Clusters 40c-42c (Fig. 5A, top right). CRE elements are known binding sites for Atf1p. Consistent with this finding, Clusters 40c to 42c were enriched for Atf1p-regulated genes (see previous section). Notably, CRE is also associated with the control of mRNA degradation during response to osmotic stress in fission yeast.^16^ The second 5′UTR/promoter motif was the Homol-D box which was enriched in Clusters 27c-29c and is known from ribosomal protein promoters.^44^ Accordingly, Clusters 27c-29c contained most ribosomal proteins (see above; Figure 5A; Table S6). Taken together, these data demonstrate the ability of the FIRE algorithm to identify known and functional sequences in our data.

**Figure:**
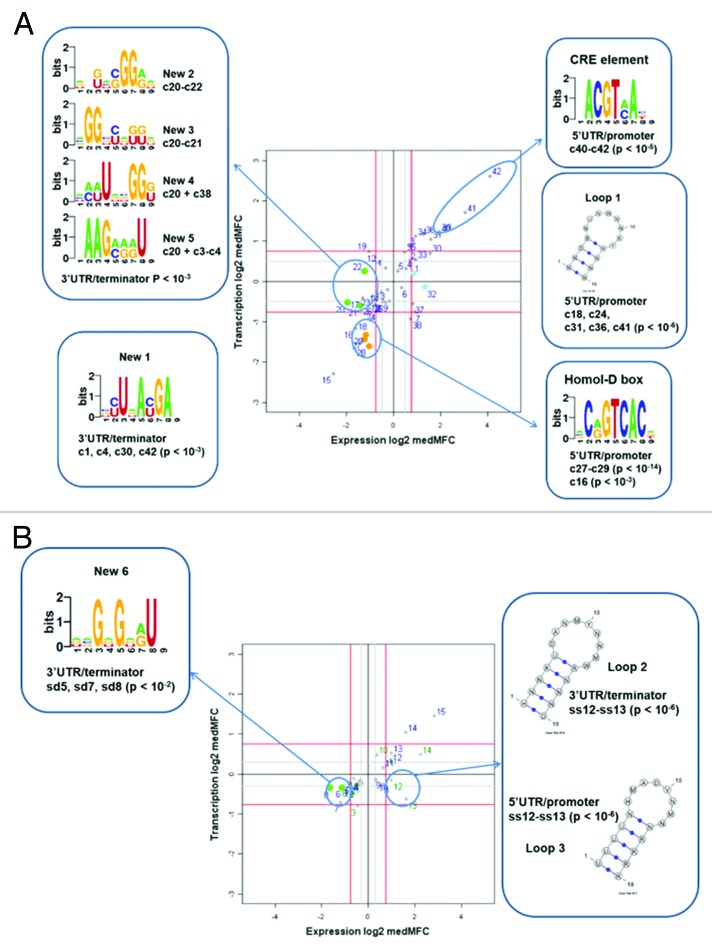
**Figure 5.** Sequence motifs associated with distinct regulatory strategies.(**A**) Clusters from the ‘constant’ model were plotted as a function of their medMFC_EXP_ and medMFC_POL_ (see legend Figure 2). The FIRE and TEISER algorithms were run using 300 nucleotide regions up- and down-stream of genes from the ‘constant’ clusters. Identified motifs (FIRE) and secondary-structure loops (TEISER) are shown together with the clusters they are enriched in. The significance p values of motif or loop enrichment in the clusters are shown in parentheses. Additional information is provided in Tables S6 and S7.(**B**) As in (**A**) but with clusters from the ‘switch’ model, both for stabilized (1ss-14ss, triangles) and destabilized (1ds-15ds, circles) clusters.

We then analyzed the 3′UTR/terminator regions of genes and identified six enriched motifs, of which five were found in clusters assigned to the ‘constant’ decay rate model and one to the ‘switch’ model. Four of them were enriched in all or some members of the 20c-22c/6sd-8sd cluster groups which are enriched for genes of the Ribi regulon, providing candidate regulatory sequences for post-transcriptional regulation of this pathway (New2-New6; Figure 5A,B; Table S6). Another motif, New1, was associated with different sets of stress induced genes regulated by Atf1p (Cluster 42c), or mostly regulated post-transcriptionally (Cluster 1c, and to lesser extent Cluster 30c). Interestingly, New1 is highly similar to the budding yeast PIN4–1 motif, which is recognized by Pin4p,^50^ an RNA-binding protein involved in cell-cycle regulation and DNA damage repair. Pin4p has two orthologous genes in fission yeast, Cip1p and Cip2p, which are both involved in post-transcriptional gene regulation during response to oxidative stress and interact with the RNA-binding protein Csx1p.^51^

Domains recognized by RNA-binding proteins can be difficult to identify because they involve complex secondary structures rather than linear sequence motifs. With this issue in mind, we also analyzed our data using TEISER, an algorithm that identifies RNA secondary structures common to groups of genes.^52^ TEISER discovered three novel structures associated with distinct transcriptional and mRNA expression signatures (Loop1-Loop3; Figure 5; Table S7). Loops 2 and 3 were found in two clusters enriched for *tf2* retrotransposons. Consistent with this finding, *tf2* elements contain duplicated regulatory sequences at their 5′ and 3′ ends. In conclusion, our analysis identified potential regulatory sequences that may participate in regulating mRNA expression levels during the response to oxidative stress.

Beside sequence motifs recognized by specific RNA-binding proteins, global RNA processing and surveillance pathways could also affect the mRNA stability during stress. For instance, pre-mRNA splicing and turnover are widely affected in response to a range of environmental conditions in budding yeast.^53^^,^^54^ RNA surveillance pathways is also linked to gene regulation during stress. For instance, a connection between the Nonsense-mediated mRNA decay surveillance pathway (NMD) and mRNA levels during oxidative stress has been observed in fission yeast.^55^^,^^56^ Moreover, NMD participates in the regulation of ribosomal protein expression during the osmotic stress response in budding yeast.^57^ These examples illustrate how global RNA processing and surveillance pathways can also serve as regulators of gene expression in response to external stimuli.

### Regulation of mRNA turnover during the adaptation to oxidative stress

During prolonged stress, the expression levels of most genes will eventually return closely to those of the steady-state condition before stress induction. This adaptation is likely to involve transcriptional control to adjust the production of transcripts, but could also involve the adjustment of mRNA turnover. To investigate this question, we selected clusters from the ‘constant’ and ‘switch’ models that showed signs of gene expression resuming pre-stress levels (Fig. S7A-C; Table S5). This analysis revealed two gene classes. Of the genes returning to pre-stress expression levels, 54% were assigned to the ‘constant’ decay rate model, while the other 46% showed adjustment of decay rates late during the stress response. This late adjustment occurred for all clusters in a small, defined time window after stress induction (35–48 min). Shalem et al.^14^ have reported global mRNA stabilization accompanying transcriptional repression during oxidative stress in budding yeast, and such late changes in mRNA turnover probably also reflect adaptation of gene expression to stress. Genes in the clusters which show signs of returning to pre-stress levels tend to be enriched for intron-less genes (Fig. S11; Table S5), independently of the decay rate model. This result is consistent with the finding that genes with rapidly regulated expression levels are intron poor.^58^ Taken together, our data indicate that adjustment of both transcription and mRNA turnover are used during recovery from oxidative stress.

### Conclusion

We applied two simple, non-invasive genomic techniques, combined with kinetic mathematical modeling, to reveal contributions of transcription and RNA turnover for the dynamic tuning of RNA levels in response to oxidative stress. While the regulation of transcription appears to be dominant, mRNA turnover is often modulated coherently with transcriptional control at the very beginning of the stress response. Intriguing information is emerging on how transcription and RNA turnover can be mechanistically coupled. The two Pol II subunits Rpb4 and Rpb7 function during transcription but also affect mRNA turnover in the cytoplasm, and they thus coordinate mRNA production with degradation,^59^^,^^60^ and even with translation.^61^ Other cytoplasmic mRNA decay factors, which shuttle between the nucleus and cytoplasm, are also required for efficient transcription and therefore link these two levels of gene regulation.^62^ Moreover, the RNA exonuclease Xrn1 is involved in a feedback mechanism between RNA synthesis and degradation which buffers transcript levels by compensating for impaired transcription or RNA turnover.^63^^,^^64^ The intricate relationship between transcription and RNA turnover goes beyond a simple coordination, and we report specialized regulatory strategies for distinct functional gene groups. These strategies include simultaneous transcriptional repression and mRNA destabilization for ribosomal protein genes, delayed mRNA destabilization with varying contribution of transcription for the ribosome biogenesis regulon, dominant roles of mRNA stabilization for genes functioning in protein degradation, and adjustment of both transcription and mRNA turnover during stress adaptation. These examples highlight the intricate, multi-faceted interplay between transcription and RNA turnover during the dynamic regulatory response to stress. We identified novel regulatory sequences that might be recognized by RNA-binding proteins and/or transcription factors and could thus contribute to the control of the different mRNA expression patterns during stress. It has been shown that regulatory elements in gene promoters play critical roles not only for transcription but also for RNA turnover.^65^^-^^67^ In fission yeast, genes regulated during oxidative stress are largely dependent on the stress-activated protein kinase Sty1p and, to a lesser extent, on the transcription factor Atf1p.^45^^,^^46^^,^^68^ We show here that genes regulated independently of Atf1p are mainly controlled by mRNA turnover. The proteins involved in this regulation remain to be identified, but our sequence motif analyses, along with published data,^51^^,^^69^ point to the RNA-binding proteins Cip1p, Cip2p and Csx1p as important players for this process. In budding yeast, the Hog1p kinase coordinates and regulates multiple levels of gene expression during the osmotic stress response,^70^ and it will be interesting to investigate whether the fission yeast ortholog Sty1p plays equally wide-ranging roles.

## Experimental Procedures

### Cell culture

Wild-type 972 h^-^ cells were grown in yeast extract (YE) medium to exponential phase at 32 °C. In order to induce oxidative stress, cells were treated with 0.5 mM H_2_O_2_, and samples for ChIP-chip and expression analysis were collected just before and at various time points after H_2_O_2_ addition (Fig. S1). One half of each sample was immediately filtered and frozen in dry ice for subsequent RNA extraction. The second half was immediately fixed in 1% formaldehyde as described.^2^

### RNA extraction and microarray expression analysis

Total RNA was extracted from each time point using the hot-phenol technique as described.^31^ Each time point was then hybridized on spotted DNA microarrays using a pool of all time points as a reference. cDNA labeling, microarray hybridization and scanning was performed as described before.^31^ Spot intensities were extracted using the GenePix Pro software and normalized using an in-house procedure.^31^ Microarray data generated in this study have been deposited in ArrayExpress with accession numbers E-MTAB-504 and E-MTAB-2579

### Pol II chromatin immunopreciptitation and microarray analysis

Pol II chromatin immunoprecipitation was performed as described^2^ using the 4H8 anti-CTD antibody (Abcam). Immunoprecipitated material for each time point was hybridized against a fraction of its respective input material (whole cell extract used as input for the immunoprecipitation) on the same microarray platform used for expression analysis. DNA labeling, microarray hybridization and scanning was performed as described.^2^^,^^31^ Spot intensities were extracted using the GenePix Pro software and normalized using an in-house procedure.^31^

### Validation time-course

Low resolution validation time courses for both expression and Pol II chromatin immunoprecipitation were generated following the procedures described above (Fig. S10).

### Modeling of mRNA expression, transcription rate and decay rate

We first assume a zero-order transcription rate and first-order decay rate, with negligible cell growth during the stress response. The time-varying absolute mRNA concentration E(t) and transcription rate R(t) for a given mRNA species satisfy:

(1) $$\frac{dE}{dt}=R(t)-kE(t)$$

where k is the decay rate constant, and half-life t_1/2_ = ln2 / k. Solving (Eq 1), mRNA concentration E(t) is given by:

$$E(t)=\int_{}^{t}R(t')e^{k(t'-t)}dt'+\text{​}E_{0}e^{-kt}$$

Transcription rates and expression values were reported relative to reference hybridizations. We therefore restate (Eq 1) to allow for unknown scaling of absolute transcription rates relative to mRNA concentration, which is present when these values are reported relative to reference hybridizations. Introducing a gene-specific scaling term (A') and shift term (B'), the measured relative expression values y(t) and relative transcription rates f(t) satisfy:

$$\frac{dy}{dt}+ky(t)=A'f(t)+\text{​}B'$$

with solution:

(2) $$y(t)=A\int_{}^{t}f(t')e^{k(t'-t)}dt'+\text{​}B+\text{​}Ce^{-kt}$$

where A, B, C are gene-specific constants. This model (Eq 2) is referred to as the 'constant' model.

To investigate whether a change in mRNA half-life can be detected during the stress time course, we consider an alternative model in which the decay rate is piecewise constant with decay rate constants k_1_, k_2_ (cf. Equation 1):

$$\frac{dE}{dt}=R(t)-k_{1}E(t);\;t\leq t_{switch}$$

$$\frac{dE}{dt}=R(t)-k_{2}E(t);\;t>t_{switch}$$

with solution after restating in terms of measured relative expression values y(t) and relative transcription rates f(t):

(3) $$y(t)=A\int_{}^{t}f(t')e^{k_{1}(t'-t)}dt'+\text{​}B+\text{​}Ce^{-k_{1}t};\;t\leq t_{switch}$$

$$y(t)=A\int_{}^{t}f(t')e^{k_{2}(t'-t)}dt'+\text{​}B\frac{k_{1}}{k_{2}}+\text{​}Ge^{-k_{2}t};\;t>t_{switch}$$

where A, B, C are gene-specific constants, and G is chosen to satisfy continuity of y(t) at t_switch_ (denoted t_s_ for brevity):

G=ek2ts(A∫tsf(t')ek1(t'−t)sdt'+​B+​Cek1ts−A∫tsf(t')ek2(t'−ts)dt'−Bk1k2)

This model (Eq 3) is referred to as the 'switch' model.

### Model fitting to observed expression values and transcription rates

Each model ('constant', Eq 2; and 'switch', Eq 3) was fitted to observed relative expression values y(t) and transcription rates f(t) independently for each gene. Parameter values were sought to minimize $\sum_{i}^{}{\left(y_{i}-{\hat{y}}_{i}\right)}^{2}$, where $y_{i},{\hat{y}}_{i}$ are observed and fitted to relative expression values, respectively. As f(t) and y(t) are in fact both measured variables, the robustness of model assignment to the addition of noise in both measured variables was investigated (Fig S6E and F). Minimization over parameters ('constant' model: A, B, C, k; 'switch' model: A, B, C, k_1_, k_2_, t_switch_) was performed using UObyQA optimization implemented in the R package ‘powell’

(http://cran.r-project.org/web/packages/powell/index.html). Initial values for the 'constant' model were A = B = C = 1, k = 1/30 min^−1^. Randomly selected initial values were tested for a subset of genes and resulted in similar reported optimal values. Initial values for the 'switch' model were A = B = C = 1, k_1_ = k_2_ = 1/30 min^−1^. Tests with randomly selected initial values for t_switch_ indicated that reported optimal values were sensitive to the initial value of t_switch_. The t_switch_ parameter space was therefore searched by initializing t_switch_ to initial values spaced at 5 min intervals and selecting the optimum over all initial values of t_switch_. The maximum number of iterations permitted for each model fit was set to 10,000. Integration was performed using an adaptive quadrature method (R function *integrate*) by linear interpolation of f(t) (R function *approx*). Since exponential approach or decay $\left(E(t)=a+\text{​}be^{-kt}\right)$ is a possible solution for mRNA abundance under both models for any observed transcription rates f(t), the 'constant' and 'switch' models were also fitted explicitly for the case A = 0.

### Model selection

Goodness-of-fit for each fitted model M is assessed using an adjusted R^2^ statistic:

$$adjR_{M}^{2}=1-\frac{SS_{err}}{SS_{tot}}\frac{n-1}{n-p-1}$$

where n = 12, the number of timepoints, and p + 1 is the number of parameters in the fitted model. Each gene is assigned to one of (1) 'switch' model, (2) 'constant' model, or (3) poor fit. A gene is assigned to the 'switch' model if it satisfies all of the following five criteria: adjR^2^_switch_ > adjR^2^_const_; adjR^2^_switch_ > 0.6; adjR^2^_const_ < 0.9; 12 min < t_switch_ < 60 min; max{k_1_/k_2_, k_2_/k_1_} > 1.4. A gene that fails these criteria is assigned to the 'constant' model if adjR^2^_const_ > 0.6, or otherwise considered as poor fit. Model selection was first performed using the A ≥ 0 'switch' model: if a gene was not assigned to the 'switch' model using A ≥ 0, the A = 0 'switch' model fit was tested.

### Exponential approach of expression to a new steady-state

Given a first-order decay process and an initial steady-state (I), then an instantaneous change in transcription rate $\left(R_{I}\to R_{F}\right)$ and/or decay rate constant $\left(k_{I}\to k_{F}\right)$ at time t_0_ results in an exponential approach to a new steady-state, solving (Eq 1):

.$$E(t)=\frac{R_{F}}{k_{F}}+\text{​}\left(\frac{R_{I}}{k_{I}}-\frac{R_{F}}{k_{F}}\right)e^{-k_{F}t}$$

Genes displaying this special case of expression behavior were identified as those genes with best fit to the 'constant' exponential model.

### Clustering of expression profiles and transcription rate profiles

Clustering of the time course microarray data was performed using SplineCluster,^38^ a model-based Bayesian agglomerative clustering algorithm to identify the number of clusters and the assignment of genes to clusters within a non-uniformly sampled time series. Clustering was performed using a prior precision for coefficients of 10^−7^.

### Specific cluster selection

In order to select clusters highly regulated in either or both expression levels or transcription rates, we calculated the median of maximum-fold change values for expression (medMFC_EXP_) and Pol II occupancy data (medMFC_POL_) for each cluster. These values are measures of the amplitude of the variations in transcript levels and Pol II occupancy in each cluster. In order to select clusters with strong regulation in both expression and transcription, we used a cut-off of 1.7 for medMFC_EXP_ and medMFC_POL_. In order to detect clusters regulated mainly at the post-transcriptional level, we used conservative cut-offs requesting a medMFC_POL_ below 1.2 and a medMFC_EXP_ above 1.7-fold. To identify clusters showing signs of reaching back to pre-stress levels, we selected clusters for which the last two time point had values lower (late downregulation) or larger (late upregulation) than the maximum or minimum values of the time course, respectively.

### Atf1p dependence analysis

Wild-type and *atf1* deletion mutant oxidative stress response time course expression profiles of a conservative list of 989 CESR genes in oxidative stress response data^45^ were hierarchically clustered. Genes whose up- or downregulation in response to 0.5mM H_2_O_2_ was lost (Atf1p-regulated) or not (Atf1p-not-regulated) in the *atf1* mutant data were manually selected from the cluster. In addition, the medMFC_EXP_ values of every cluster described in this study were calculated for the wild-type and *atf1* mutant time courses. Clusters which significantly overlapped the ‘Atf1p-regulated’ list and had a ratio in medMFC_EXP_ over 1.5-fold in wild-type and *atf1* mutant time courses were called ‘Atf1p-dependent’. Clusters which significantly overlapped the ‘Atf1p-not-regulated’ list and had a ratio in medMFC_EXP_ below 1.5-fold between wild-type and *atf1* mutant time courses were called ‘Atf1p-independent’. Clusters which did not satisfy those criteria were not assigned. The Atf1p target list based on ChIP-chip data was from Table S1 in reference 47.

## Supplementary Material

Additional material

## Abbreviations:

Pol II: RNA Polymerase II
H_2_O_2_: hydrogen peroxide
MFC_POL_: maximum fold change in Pol II occupancy
MFC_EXP_: maximum fold change in transcript expression
TSS: transcription start site
Ribi: ribosome biogenesis
UTR: untranslated region

## Notes

10.4161/rna.29196
