# Supplementary material for: Contributions of transcription and mRNA decay to gene expression dynamics of fission yeast in response to oxidative stress
Source: RNA Biol. 2014 Jul 9;11(6):702–14. doi: 10.4161/rna.29196 (PMC4156502; doi:10.4161/rna.29196)
Supplement: Additional material [file rna-11-702-s01.pdf]

Landes Bioscience
www.landesbioscience.com

Supplemental Material to:

Samuel Marguerat, Katherine Lawler, Alvis Brazma, and Jürg Bähler

Contributions of transcription and mRNA decay to gene expression dynamics of fission yeast in response to oxidative stress

2014; 11(6)
http://dx.doi.org/10.4161/rna.29196

www.landesbioscience.com/journals/rnabiology/article/29196/

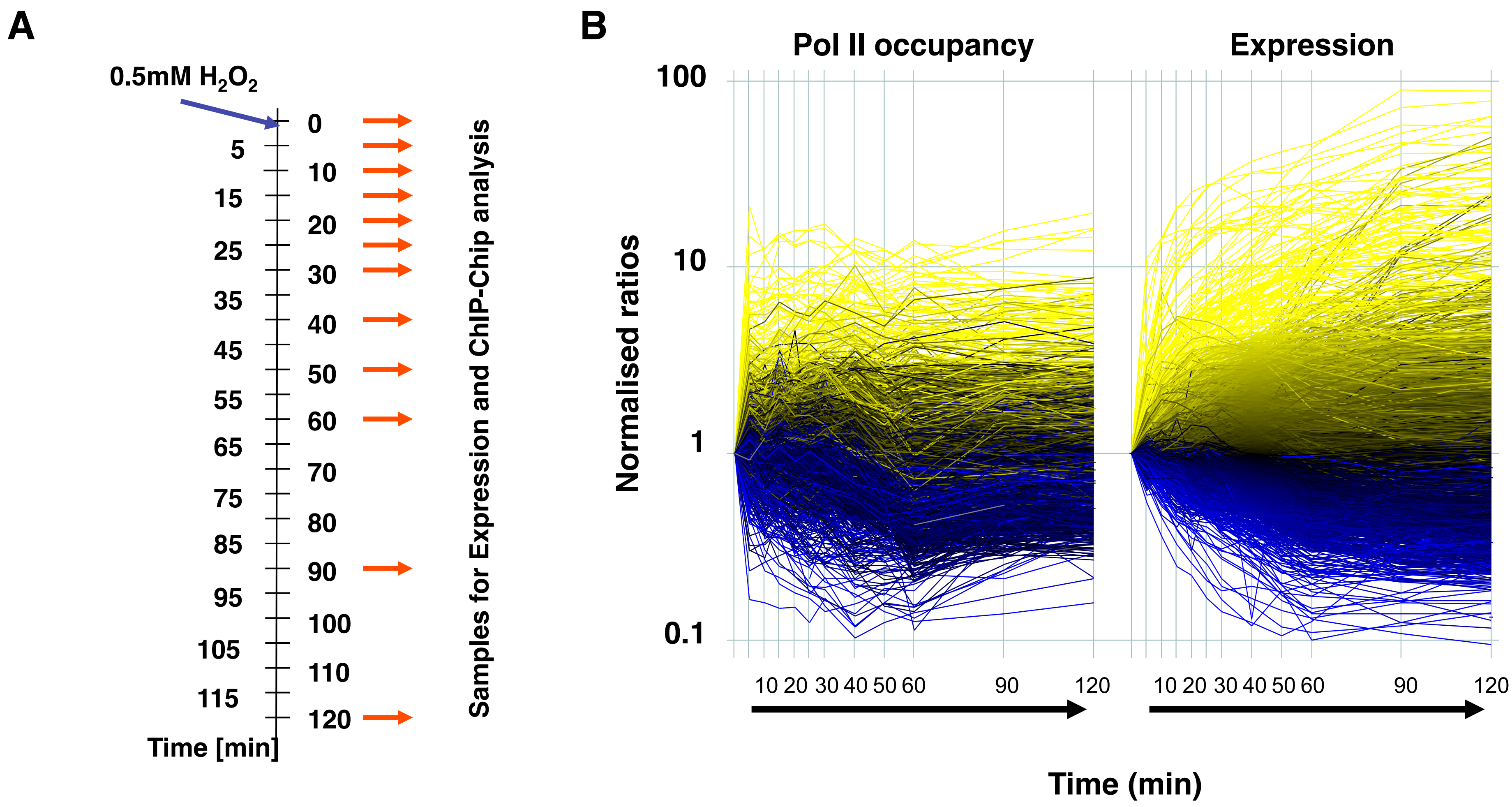

Figure S1: Experimental design.
(A) Samples were collected at various time points after oxidative stress induction (0.5mM H2O2), both for RNA Pol II ChIP-chip and transcript expression analyses.
(B) Global view of Pol II occupancy and mRNA expression profiles.

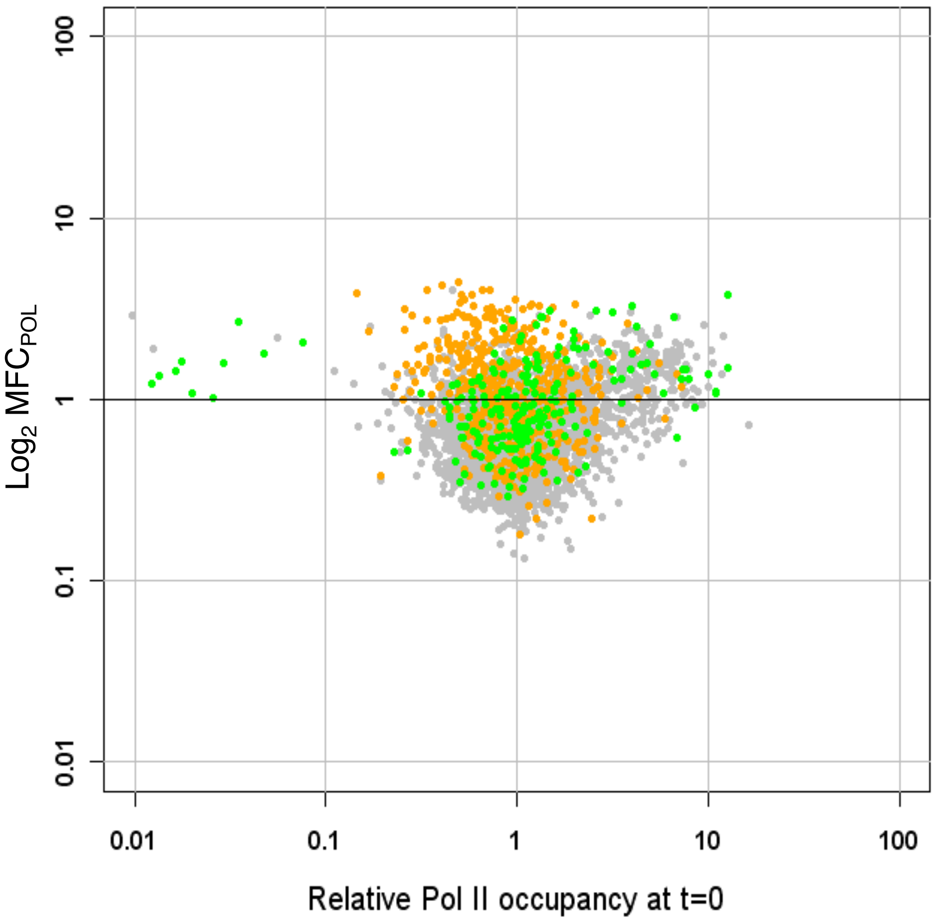

Figure S2: Changes in Pol II occupancy can be measured across the entire dynamic range.
Increase in Pol II occupancy above 2 fold can be detected independently of initial Pol II levels. Maximum Fold Changes in Pol II occupancy (MFC_{POL}) is defined as the ratio between any time points with the highest and the lowest Pol II occupancy values. Genes with strongly induced Pol II occupancy during stress are shown as orange dots, while genes with strongly reduced Pol II occupancy during stress are shown as green dots.

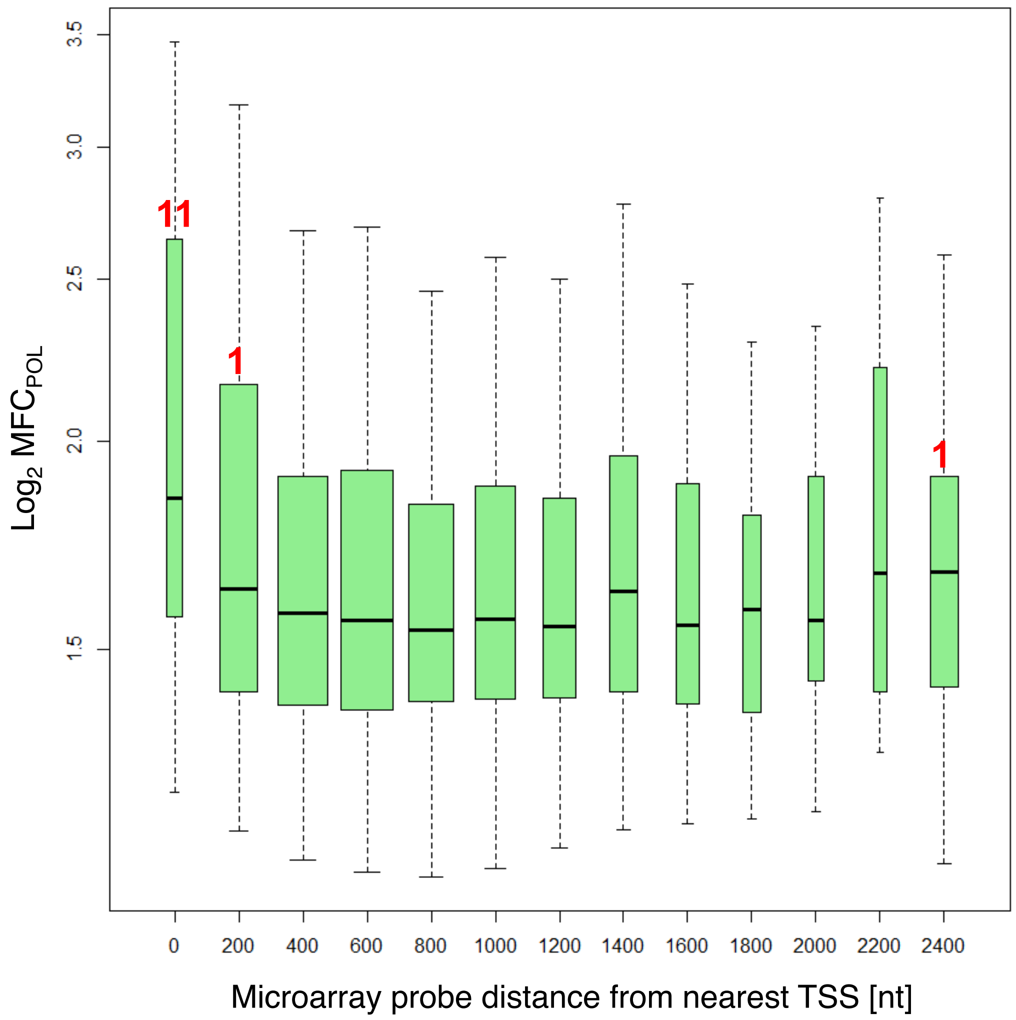

Figure S3: Changes in Pol II occupancy as function of microarray probe distance from nearest transcription start sites (TSS). Distances of microarray probes from nearest transcription start sites (TSS) were split in 13 bins of 200nt (lower bin limit is indicated in the figure), and the distribution in MFC_{POL} was plotted for each bin. If a bin has significantly higher MFC_{POL} than any other bin, the number of bins with lower MFC_{POL} than itself is represented by a red number ($p_{wilcoxon} < 0.05$).

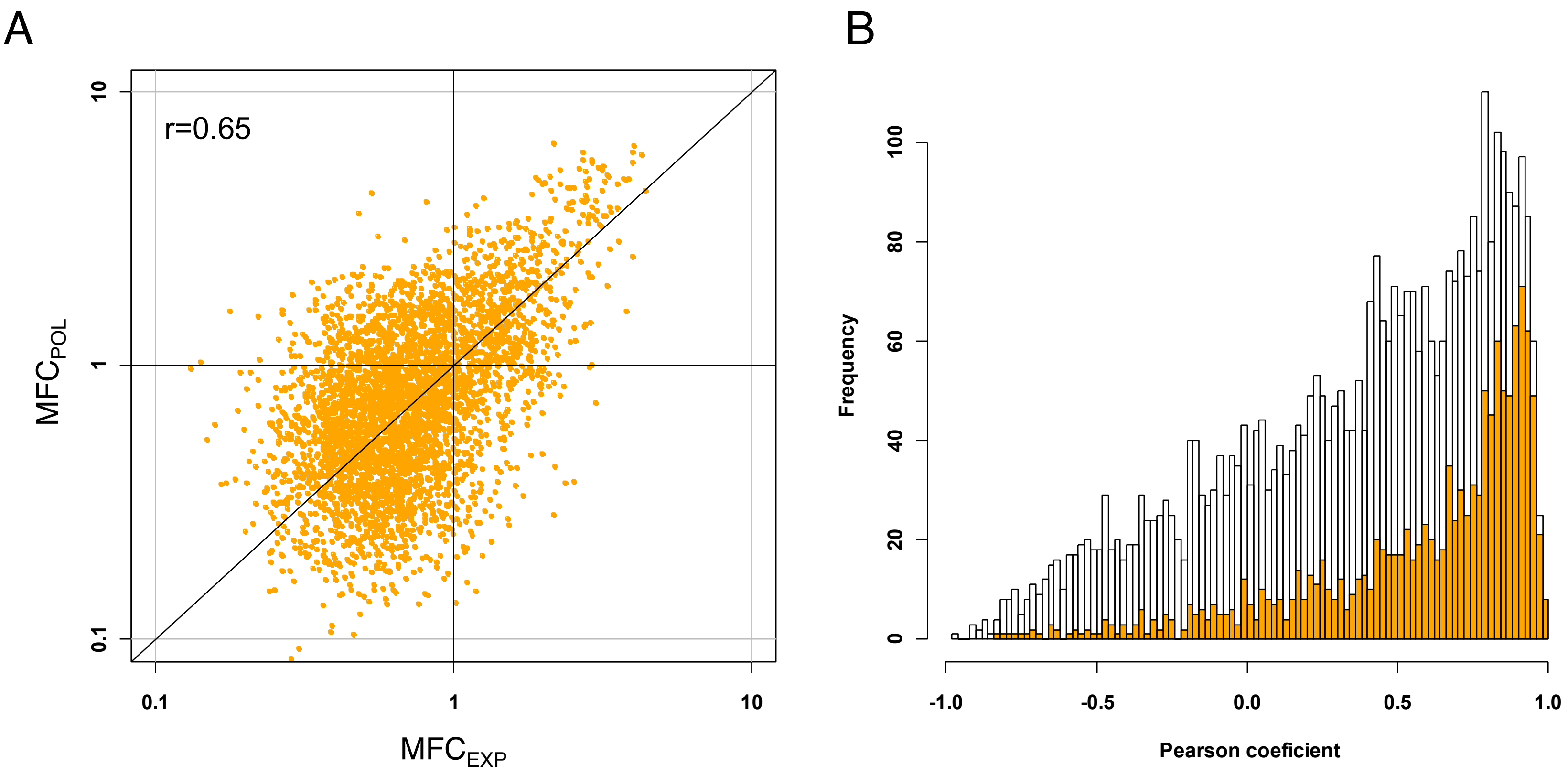

Figure S4: Changes in gene expression and Pol II occupancy are positively correlated.
(A) MFC_{EXP} and MFC_{POL} for each gene are plotted to show positive correlation ($r_{pearson}$ = 0.65).
(B) Distribution of Pearson correlation coefficients between transcript expression and Pol II occupancy for each gene (white sbars) or for genes with MFC_{EXP} >2 (orange bars).

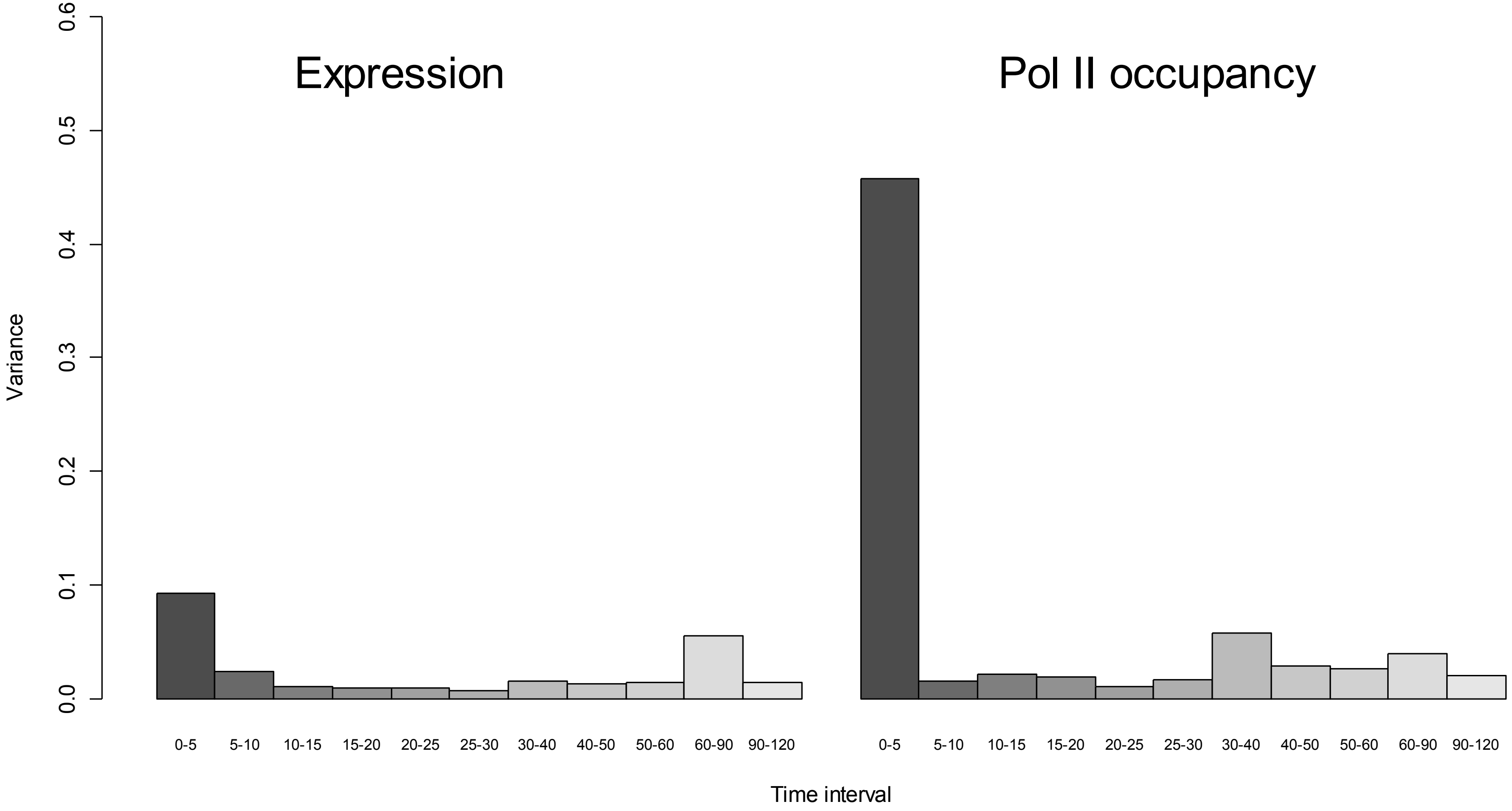

Figure S5: Most variability in Pol II occupancy occurs during the first minutes of time course. Variance of the changes in expression and Pol II occupancy was measured and plotted for all intervals between neighbouring time points.

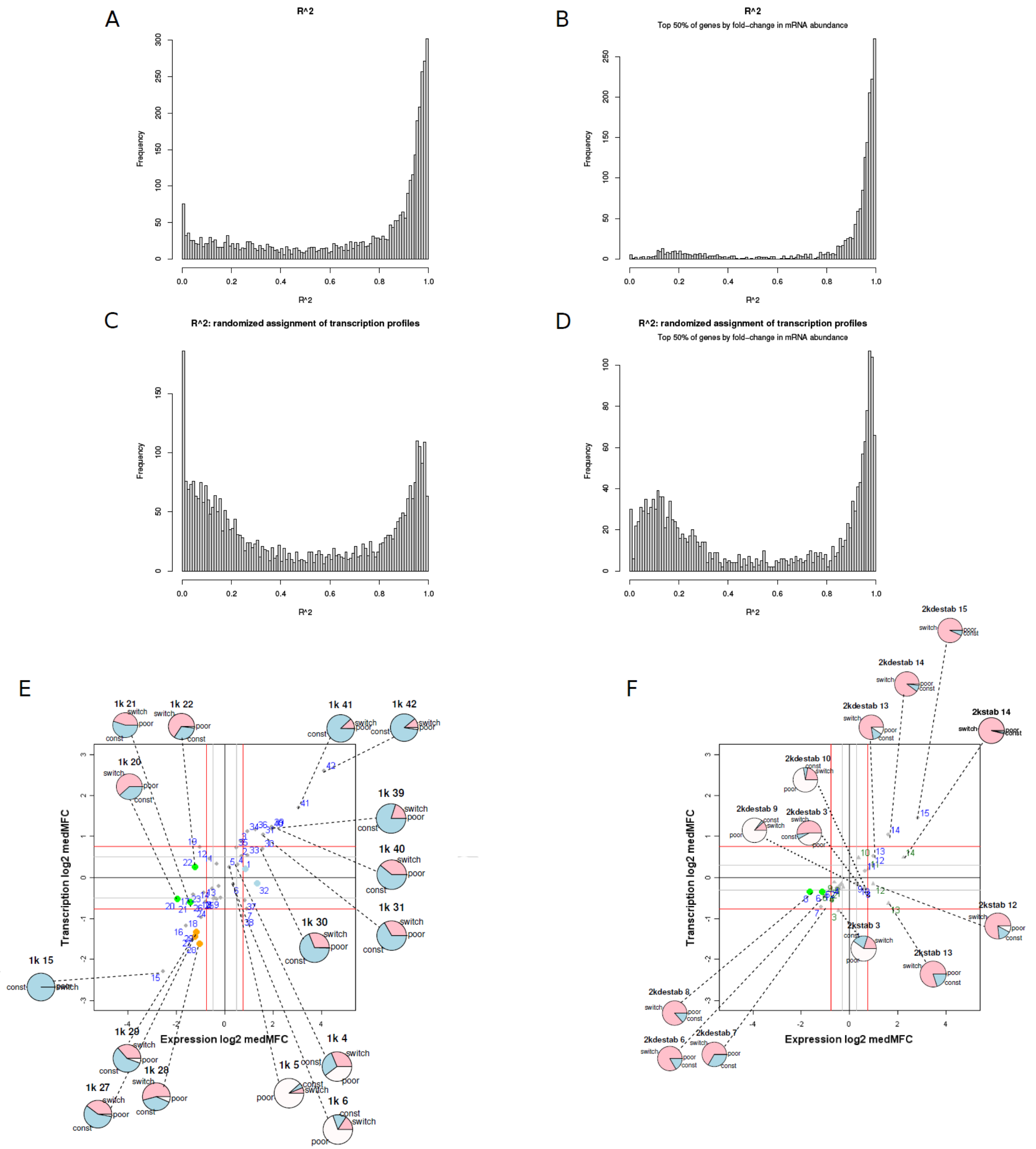

Figure S6: Exploring robustness of model assignments.
Permuting the assignment of transcription profiles to genes results in poorer model fits overall, indicating that the 'constant' model is an informative initial model. The robustness of assignment to the alternative 'switch' model was investigated by adding randomly generated Gaussian noise to standardized abundance and transcription profiles for a subset of genes.

(A) Distribution of goodness-of-fit of the constant model (shown as *max[0, R-squared]*) for all genes.

(B) As for (A), but only top 50% of genes by mRNA abundance fold-change.

(C) Distribution after permuting the assignment of transcription profiles to genes.

(D) As for (C), but only top 50% of genes by mRNA abundance fold change.

(E) The effect of adding noise (std dev = 0.1, resampled 5 times) to abundance and transcription profiles of a subset of genes in the 'constant' model clusters 1c-42c. Pie charts illustrate the proportion of model fits to constant decay rate (blue, 'const'), delayed change in decay rate (pink, 'switch'), or no fit (white, 'poor') for example clusters of interest.

(F) As for (E), but for 'switch' model clusters 1ss-14ss and 1sd-15sd, and illustrated for clusters of interest.

A

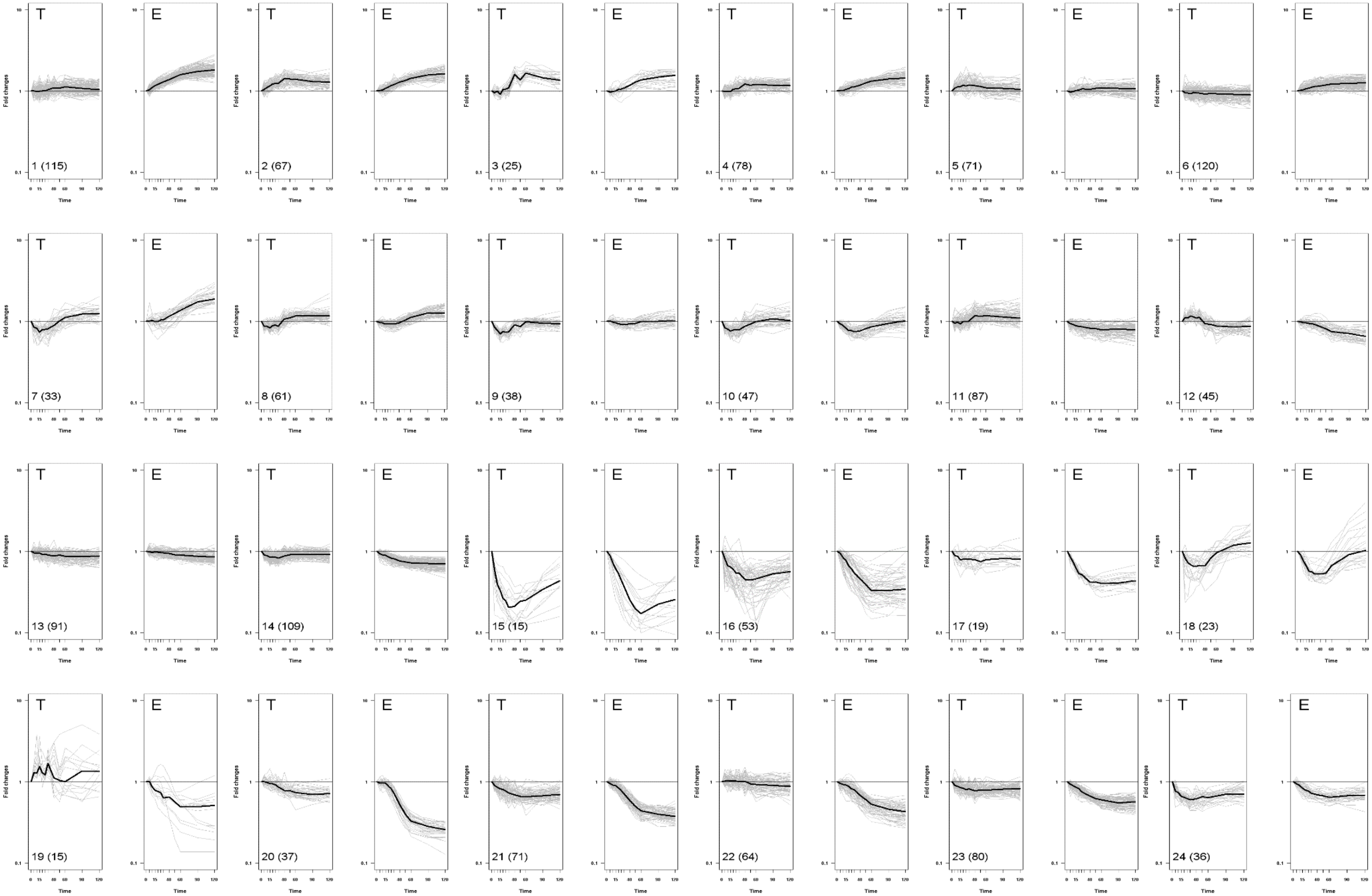

T
E
1 (115)
2 (67)
3 (25)
4 (78)
5 (71)
6 (120)
7 (33)
8 (61)
9 (38)
10 (47)
11 (87)
12 (45)
13 (91)
14 (109)
15 (15)
16 (53)
17 (19)
18 (23)
19 (15)
20 (37)
21 (71)
22 (64)
23 (80)
24 (36)
Fold changes
Time

Figure S7

A (CONTINUED)

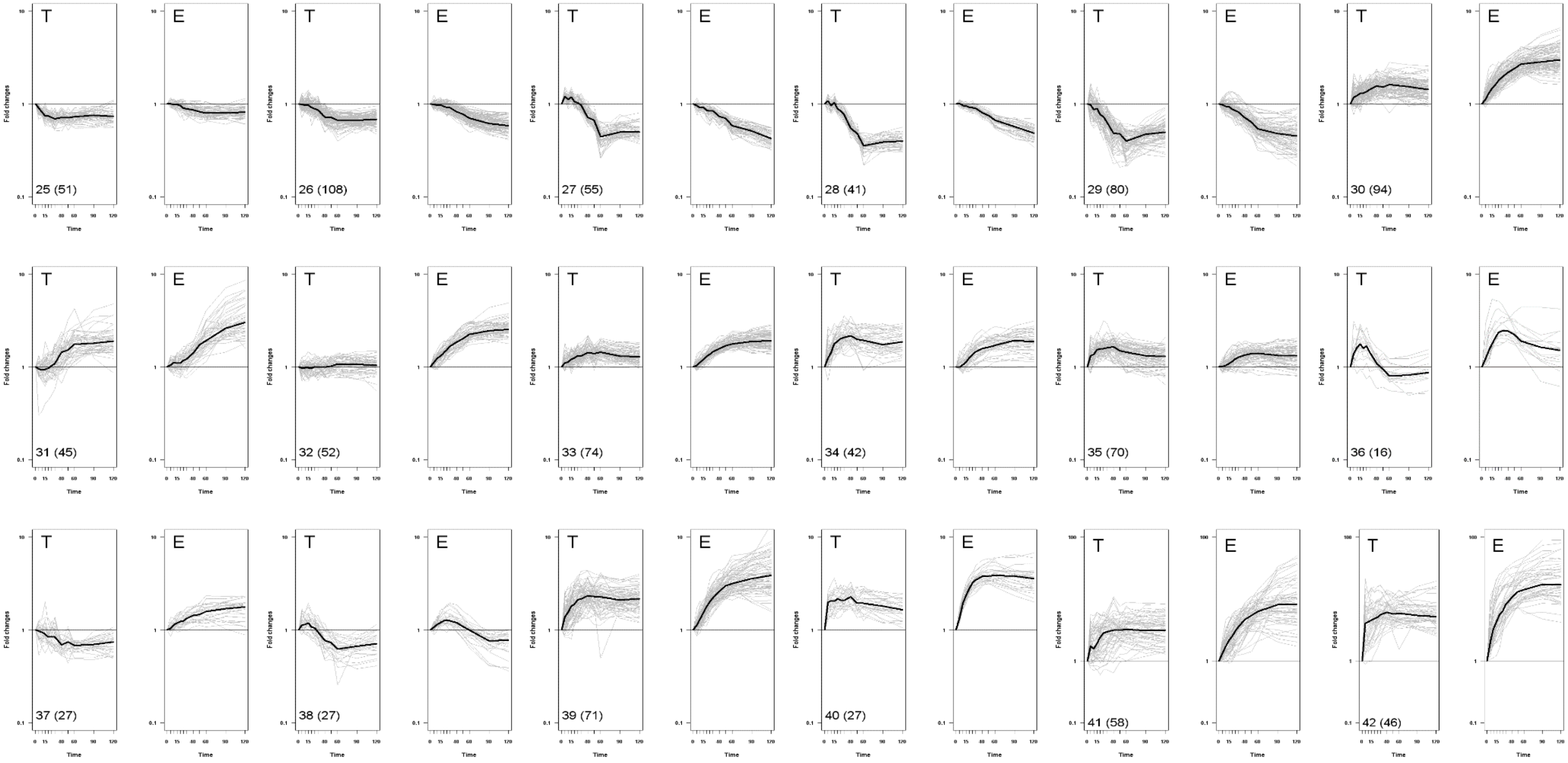

Figure S7

B

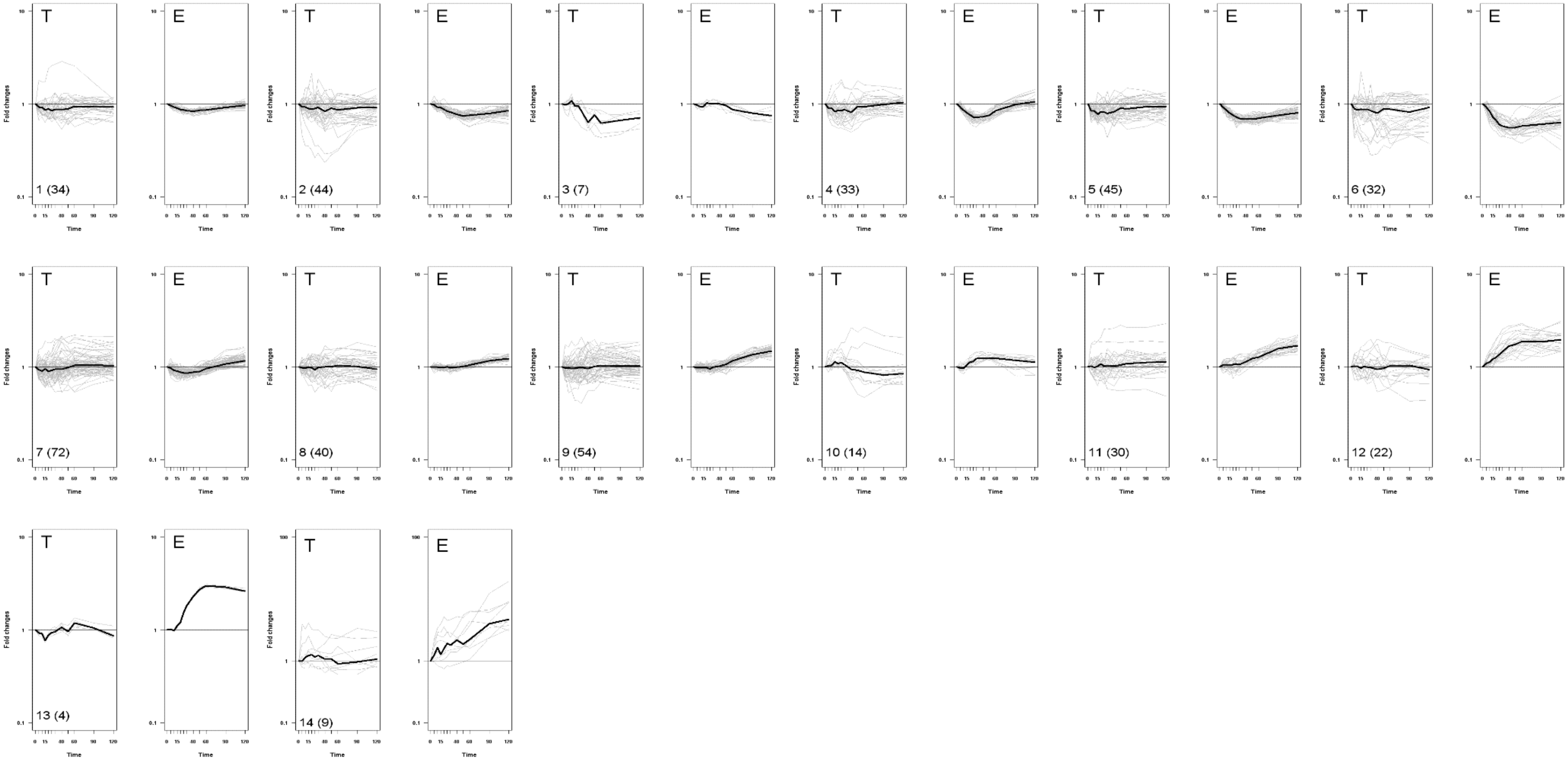

Supplementary Figure S7

C

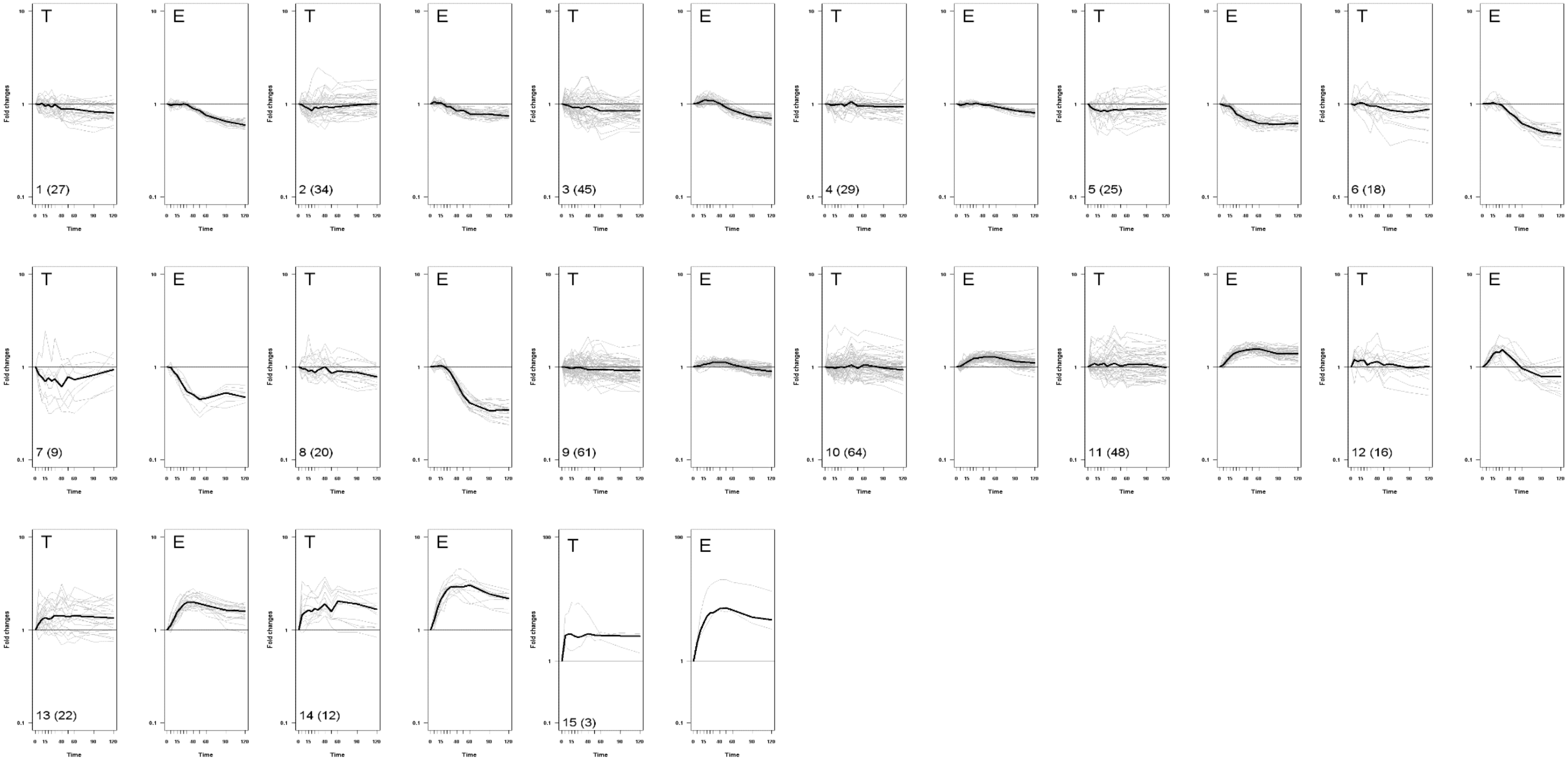

Supplementary Figure S7

D

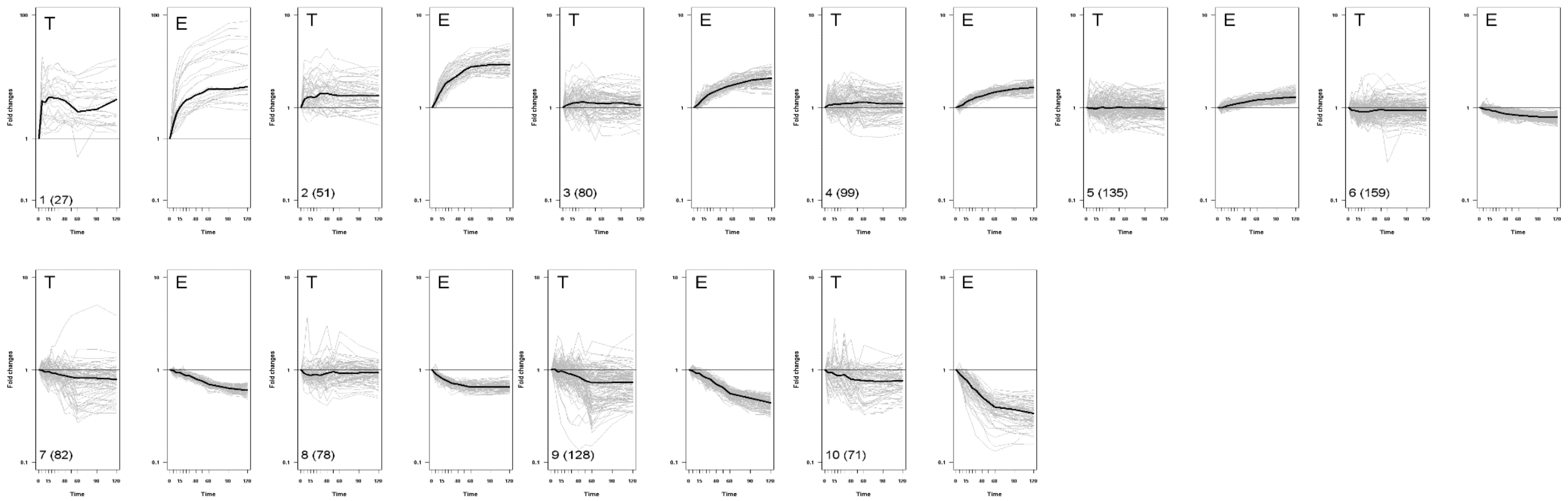

E

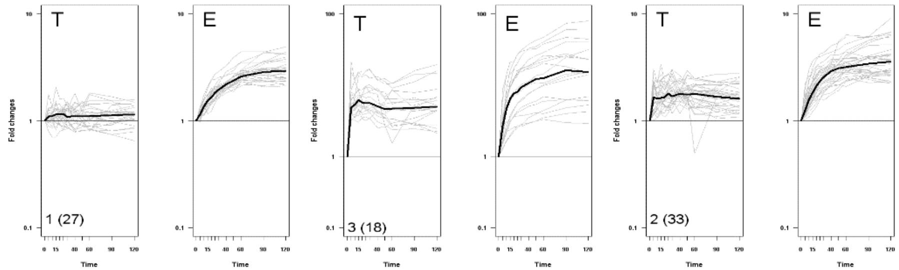

Figure S7: Clusters obtained after classification of the genes assigned to the different mathematical models. Transcription (T) and expression (E) profiles are shown for each gene (light grey) and for the median profile of each cluster (black). The numbers at bottom left indicate the cluster number and, in parentheses, the number of genes included in the cluster.

(A) Clusters from 'constant' model (1c-42c).

(B) Clusters from 'switch' model and stabilized (1ss-14ss).

(C) Clusters from 'switch' model destabilized (1sd-15sd).

(D) Genes showing an exponential approach to new steady state (1e-10e).

(E) As D re-clustered (see main text).

Figure S7

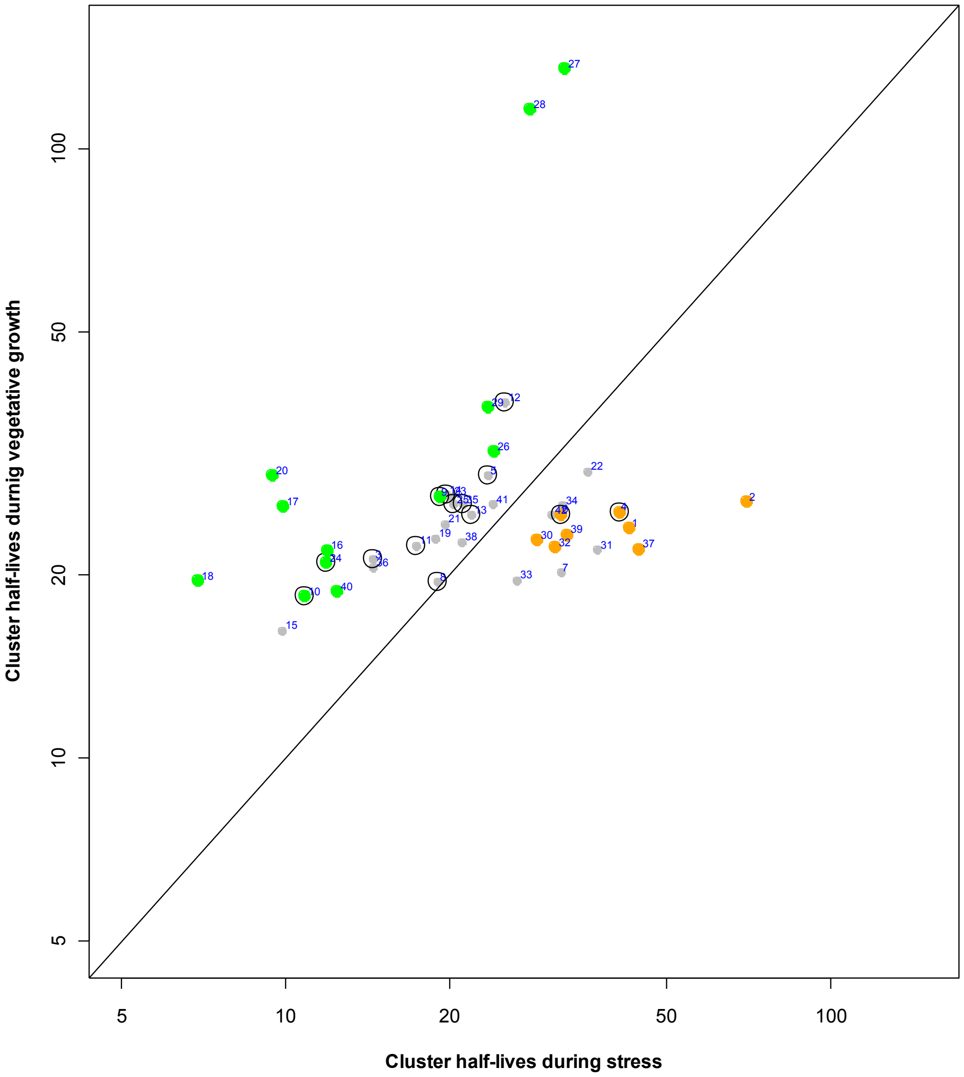

Figure S8: Comparison of mRNA stability before and after stress induction. mRNA half-lives measured in unstressed cells (from reference 34) were compared to mRNA half-lives during the stress response derived from mathematical modelling. Clusters from the ‘constant’ model are plotted as a function of half-lives before and after stress induction. Green dots show clusters with significantly shorter half-lives during stress, while orange dots show clusters with significantly longer half-lives during stress (P_{wilcox} <0.01). Clusters with small $\log_2$ med MFC_{EXP} (between -0.7 and 0.7) are circled in black.

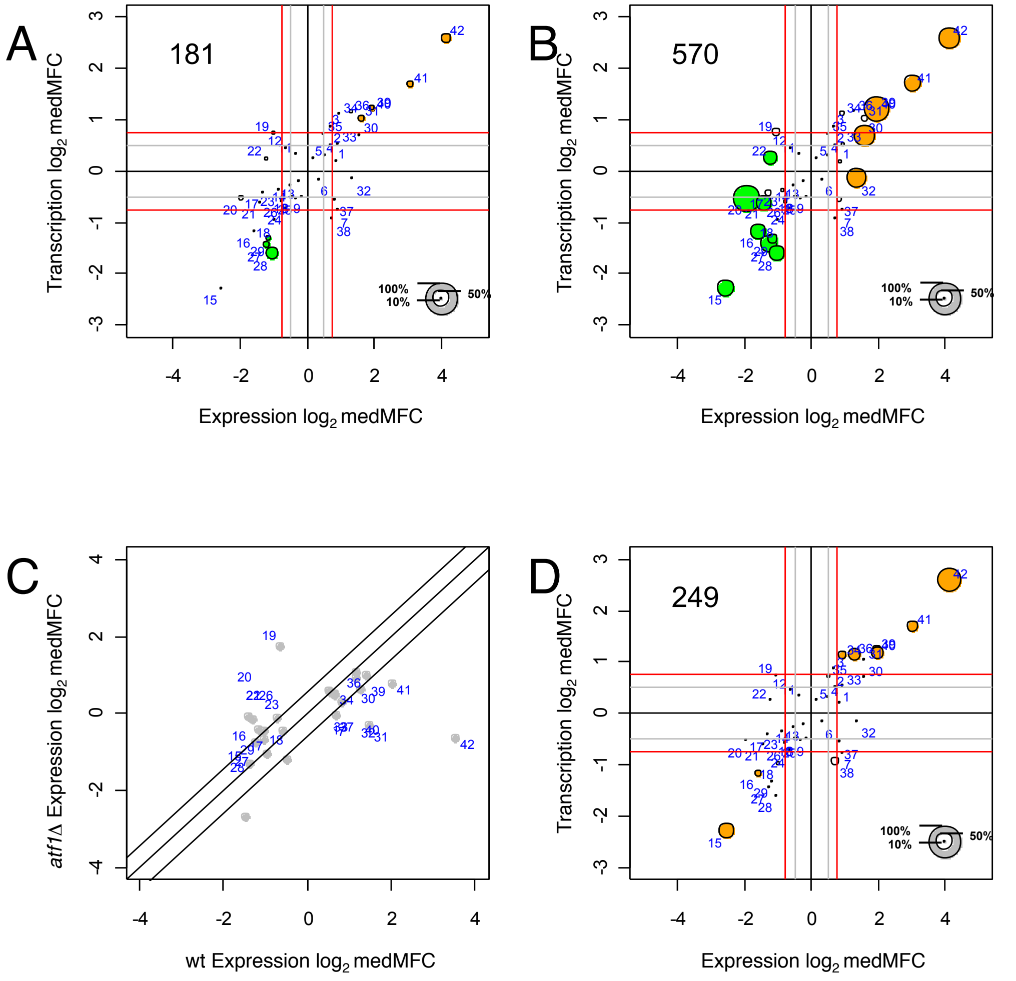

Figure S9. Analysis of Atf1p-dependent and Atf1p-independent clusters.

(**A**) Clusters from 'constant' model were plotted as a function of their $medMFC_{EXP}$ and $medMFC_{POL}$ (see legend Fig. 2), and overlap of each cluster with the 'Atf1p-regulated' gene list was measured (Experimental procedures). The size of the circle is proportional to the percentage of genes from each cluster present in the list as indicated in legend at bottom right. Clusters colored in orange (up-regulated) or green (down-regulated) indicate significant overlaps (P_{Fisher} <0.001).

(**B**) As in (A), but with the 'Atf1p-not-regulated' list (Experimental procedures).

(**C**) Clusters from the 'constant' model were plotted as a function of their $medMFC_{EXP}$ during the oxidative stress response in wild-type or *atf1* mutant cells[41]. Lines depict 1.5-fold change.

(**D**) As in (A), but with a list of 251 Atf1p target genes determined by ChIP-chip[61].

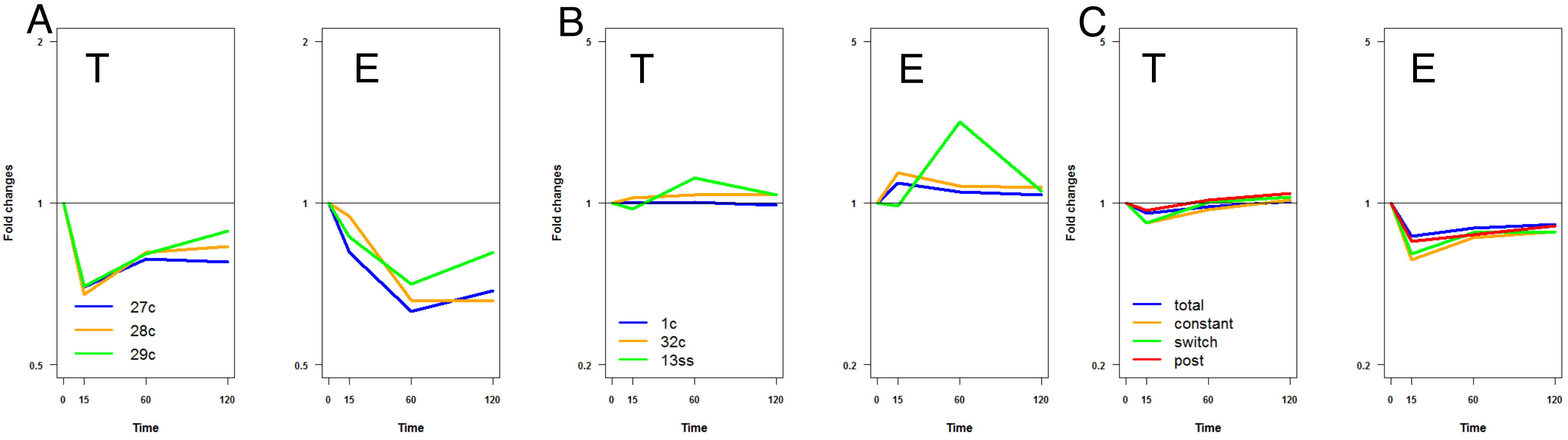

Figure S10: Specific gene categories use distinct expression strategies.
Median transcription (T) and expression (E) profiles of selected gene groups with common expression strategies are plotted as in Fig. 3 but using data of an independent low resolution validation time course. Names of gene groups are indicated next to the coloured lines.
(A) Median profiles of ribosomal proteins distributed in three clusters from the ‘constant’ model.
(B) Median profiles of three clusters showing regulation mostly at the mRNA degradation level. Clusters 1c and 32c contain genes related to protein degradation, while Cluster 13ss in enriched for *tf2* elements.
(C) Median profiles of four subcategories of genes from the Ribi regulon: ‘total’, all genes in the list; ‘constant’, genes assigned to the ‘constant’ model showing transcriptional regulation; ‘switch’, genes assigned to the ‘switch’ model; ‘post’, genes from the ‘constant’ model showing little or no regulation at the transcriptional level.

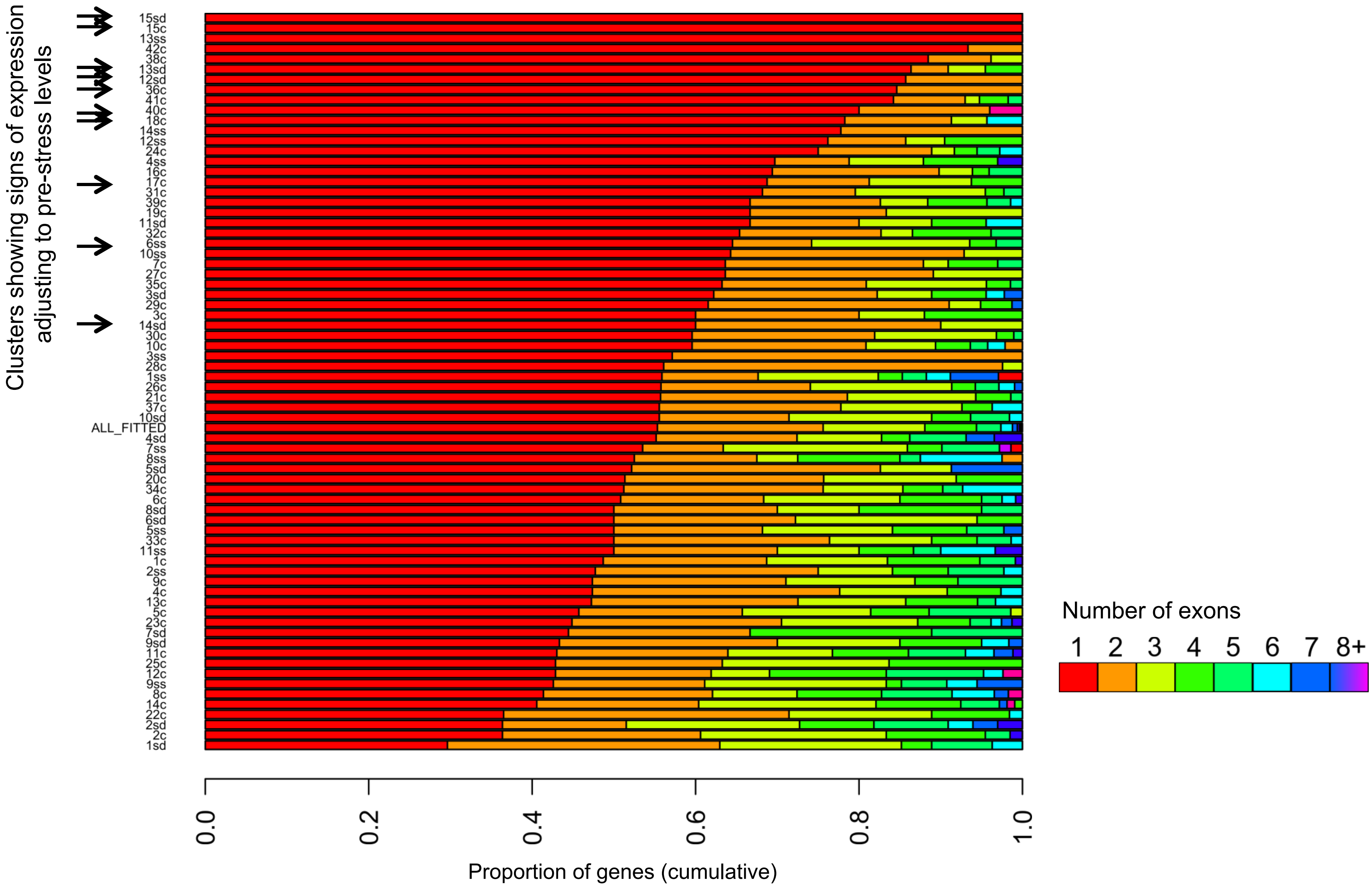

Figure S11. Distribution of exon numbers for each cluster. The distribution of exon numbers is shown for each cluster in Fig S7A-C (c: constant model; ss: switch, stabilized; sd: switch, destabilized; 'ALL_FITTED': all clusters combined, representing the background distribution of exon numbers). Clusters are ordered by the proportion of intron-less genes (one exon). Arrows indicate the clusters which show signs of expression adjusting to pre-stress levels (Table S5).
